# Improved human disease candidate gene prioritization using mouse phenotype

**DOI:** 10.1186/1471-2105-8-392

**Published:** 2007-10-16

**Authors:** Jing Chen, Huan Xu, Bruce J Aronow, Anil G Jegga

**Affiliations:** 1 Division of Biomedical Informatics, Cincinnati Children's Hospital Medical Center, Cincinnati, USA; 2 Department of Biomedical Engineering, University of Cincinnati, Cincinnati, USA; 3 Department of Pediatrics, University of Cincinnati College of Medicine, Cincinnati, USA

## Abstract

**Background:**

The majority of common diseases are multi-factorial and modified by genetically and mechanistically complex polygenic interactions and environmental factors. High-throughput genome-wide studies like linkage analysis and gene expression profiling, tend to be most useful for classification and characterization but do not provide sufficient information to identify or prioritize specific disease causal genes.

**Results:**

Extending on an earlier hypothesis that the majority of genes that impact or cause disease share membership in any of several functional relationships we, for the first time, show the utility of mouse phenotype data in human disease gene prioritization. We study the effect of different data integration methods, and based on the validation studies, we show that our approach, ToppGene , outperforms two of the existing candidate gene prioritization methods, SUSPECTS and ENDEAVOUR.

**Conclusion:**

The incorporation of phenotype information for mouse orthologs of human genes greatly improves the human disease candidate gene analysis and prioritization.

## Background

Although the availability of complete genome sequences and the wealth of large-scale biological data sets opened up unprecedented opportunities to elucidate the genetic basis of rare and common human diseases [1], comprehending the underlying pathophysiological mechanisms continues to be challenging. Majority of the common diseases are genetically intricate, polygenic and multifactorial, and frequently manifest as different clinical phenotypes. Additionally, these complex conditions are often triggered by an interaction of genetic, environmental, and physiological factors, making it difficult for researchers to narrow their focus to a single or few genes. High-throughput genome-wide studies like linkage analysis and gene expression profiling although useful for classification and characterization do not provide sufficient information to identify specific disease causal genes. Both of these approaches typically result in hundreds of potential candidate genes, failing to help the researchers in reducing the target genes to a manageable number for further validation.

Functional enrichment approaches [2-4] focusing on gene sets that share common biological function, chromosomal location, or regulation although successful in identifying enriched biological themes are not suitable for gene prioritization. To overcome this, several gene prioritization methods have been developed [5-9] (see Tiffin et al [10] and Oti and Brunner [11] for a complete list of existing approaches and web tools for the prediction or prioritization of disease candidate genes). POCUS [6], for instance, finds candidate genes by identifying an enrichment of keywords associated with gene ontology (GO), shared protein domains and expression profiles among a given set of susceptibility loci relative to the genome at large. Similarly, PROSPECTR [8] and SUSPECTS [12], focusing on Mendelian and oligogenic disorders, compare GO, protein domains and expression libraries of putative disease genes with those known to be involved with the same disease. Integrating genomic and proteomic data, Mootha et al [13] identified LSFC (Leigh syndrome, French-Canadian type) causal gene. The recent method, ENDEAVOUR [9], uses several data sources to prioritize candidate genes. None of these approaches however utilize the mouse phenotype data in their prioritization approaches although mouse is the key model organism for the analysis of mammalian developmental, physiological and disease processes [14]. Additionally, there have been several reports [15,16] wherein a direct comparison of human and mouse phenotypes allowed for the rapid recognition of disease causal genes.

Extending on the above mentioned approaches, and an earlier hypothesis, that the majority of disease causal genes are functionally closely related [6], we reasoned that an integrative genomics-transcriptomics-phenomics-bibliomics approach utilizing the available human gene annotations, mouse phenotype data and literature co-citations of genes will expedite human complex disease candidate gene identification and prioritization. We call our prioritization method ToppGene (acronym for Transcriptome Ontology Pathway PubMed based prioritization of Genes). For the first time, we incorporated the mouse phenotype data as one of the feature parameters apart from GO, pathways, biomedical literature, protein domains, protein interactions and gene expression of genes to prioritize human disease candidate genes and demonstrate its utility.

## Results

### Mouse phenotype as a feature for candidate gene prioritization

The Mammalian Phenotype (MP) Ontology enables robust annotation of mammalian phenotypes in the context of mutations, quantitative trait loci and strains that are used as models of human biology and disease. The MP Ontology (MPO) supports different levels and richness of phenotypic knowledge and flexible annotations to individual genotypes [17]. Each node in MPO represents a category of phenotypes and each MP ontology term has a unique identifier, a definition, synonyms, and is associated with gene variants causing these phenotypes in genetically engineered or mutagenesis experiments. In the current study, we retrieved mouse genes associated with each of the MP term and extracted the corresponding human orthologous genes. In the current version of MPO, there are 4280 terms associated to 4329 unique Entrez mouse genes (extrapolated to 4329 orthologous human genes). We do not check whether the human orthologous gene of a mouse gene causes similar phenotype. Rather, we assume that orthologous genes cause "orthologous" phenotype and test the potential of the extrapolated mouse phenotype terms as a similarity measure between the training and test group of genes in candidate gene analysis.

### Document identifier as a feature for candidate gene prioritization

We use biomedical literature abstract identifiers (PubMed identifiers, PMIDs) as a feature for classification, where the dimensionality of the feature space was equal to the number of documents in the document set. We hypothesized that if a PMID is cross-referenced in two genes, the two genes are likely to have a direct or indirect association. A large number of co-citations for a pair of genes (i.e. same PMIDs associated with two different genes) probably represents a relationship (direct or indirect association) between the two genes. For each gene, ToppGene considers all associated articles (represented as PMIDs) as literature annotation of this gene. The gene to PMID association file ("gene2pubmed.gz") was downloaded from NCBI Entrez Gene ftp site [18]. 44806 PMIDs were associated with more than one gene and 25294 genes had at least one PMID association. 24273 genes shared at least one PMID with another gene. For the current study, we do not look into the details of the relationship type between the genes but consider only co-citation. In other words, the PMIDs are used only as a feature of similarity measure in the candidate gene analysis.

### Comparison of ToppGene with other gene prioritization approaches

To evaluate the performance of our approach and also compare it with other similar gene prioritization approaches [8,9,12], we performed two types of comparisons: large-scale cross-validations and small-scale test cases (See Additional file 1 for the workflow, and Tables 1 and 2 for a comparison of features and methods used in the 3 applications, namely, SUSPECTS, ENDEAVOUR and ToppGene). For large-scale cross-validations, we used the same or similar training sets as mentioned in the previous methods. Specifically we compared ToppGene's performance with ENDEAVOUR [9] using random-gene cross-validation; and for comparison with PROSPECTR [8] and SUSPECTS [12], we used locus-region cross-validation. Additionally, as test cases, we selected two diseases, congenital heart defects (CHD) and diabetic retinopathy (DR), and compared the prioritization performance of ToppGene with SUSPECTS [12] and ENDEAVOUR [9].

**Table 1:** Comparison of features used in the three gene prioritization applications

**Feature type**	**SUSPECTS**	**ENDEAVOUR**	**ToppGene**
Sequence Features & Annotations	Gene length Homology Base composition	Blast *cis*-element Transcriptional motifs	Not used
Gene Annotations	Gene Ontology	Gene Ontology	Gene Ontology Mouse Phenotype
Transcript Features	Gene expression	Gene expression EST expression	Gene expression
Protein Features	Protein domains	Protein domains Protein interactions Pathways	Protein domains Protein interactions Pathways
Literature	Not used	Keywords in abstracts	Co-citation (PMIDs)

**Table 2:** Comparison of methods used in the three gene prioritization applications

**Data type**	**SUSPECTS**	**ENDEAVOUR**	**ToppGene**
Attribute-based data	Semantic similarity	*p-value *from meta-analysis	Fuzzy measure based similarity
Vector-based data	Pearson correlation	Pearson correlation	Pearson correlation
Combination of scores	Weighted mean	*p-value *from order statistics	*p-value *from meta-analysis

### Comparison of ToppGene with ENDEAVOUR: Random-gene cross-validation

In the current study we used our own disease training sets because the complete data sets used by ENDEAVOUR are not available for public access. We, therefore, randomly selected 19 diseases along with their associated genes from Online Mendelian Inheritance in Man (OMIM) and the Genetic Association Database (GAD). Each disease gene set contained 30 to 44 genes. The total number of genes across 19 selected diseases was 693 (See Additional file 2 for the complete list of the datasets). For negative controls, 20 sets, each containing 35 random genes, were created as training data. We followed the same methodology as ENDEAVOUR to evaluate the performance of our prioritization method and also compare the results with ENDEAVOUR. In each validation run, the gene group of a particular disease (with one gene removed as the "target") was used as the training set. The "target" gene was then mixed with 99 *random *genes to make a test set of 100 genes. The rank of the "target" gene in the resulting list, following prioritization, was recorded. This process was repeated for each gene in the list. Sensitivity was defined as the frequency of "target" genes that are ranked above a particular threshold position, and specificity as the percentage of genes ranked below the threshold. For instance, a sensitivity/specificity value of 70/90 indicates that the correct disease gene (the "target") is ranked among the best-scoring 10% of genes in 70% of the prioritizations. Receiver operating characteristic (ROC) curves were plotted based on the sensitivity/specificity values and area under curve (AUC) was computed as the standard measure of the performance of the method. ENDEAVOUR reported 90/74 sensitivity/specificity value and an AUC score of 0.866 [9].

**Figure 1:**
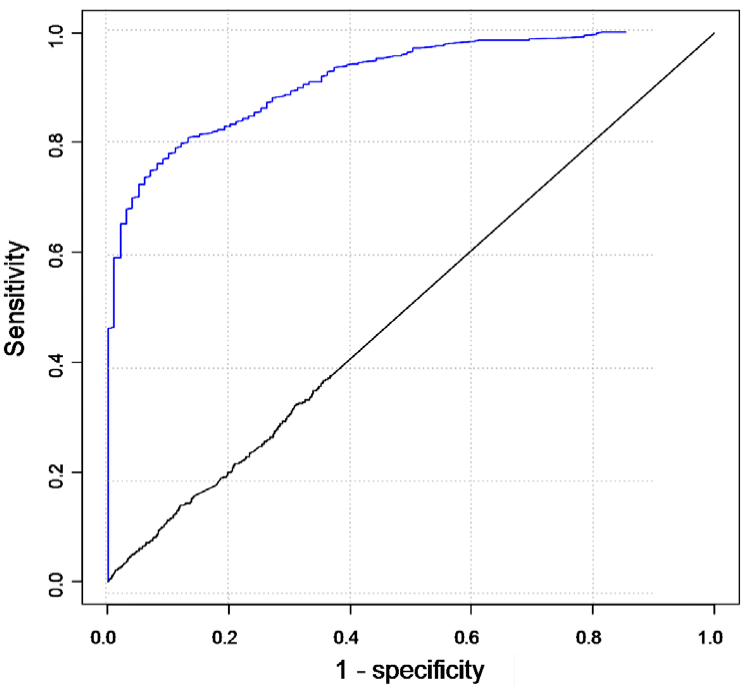
ROC curves of random-gene cross-validation based on score ranks. Blue curve was generated from the 19 disease gene training sets. Black curve, negative control, was generated from 20 random training sets. See text for the definitions of sensitivity and specificity.

Using ToppGene, we first created the overall ROC curves. In order to compare with ENDEAVOUR directly, we followed the same definitions for sensitivity and specificity as described by Aerts et al [9]. Figure 1 shows the overall ROC curves using ToppGene. The AUC score of the 19 disease training sets was 0.916, and the sensitivity/specificity was 90/77, i.e. the "target" gene was ranked among the top 23% in 90% of the cases. In case of the control, the AUC score of the 20 random training sets was 0.503 (see section A of Table 3).

**Table 3:** Summary of comparison of results from ToppGene with other gene prioritization applications

**A. Random cross-validation**			
	**ENDEAVOUR**		**ToppGene**

AUC (area under curve)	86.6		91.6
True positive rate/false positive rate	74/90		77/90

**B. Locus region cross-validation**			

	**PROSPECTR**	**SUSPECTS**	**ToppGene**

Percentage of top 5% ranked target genes	13% (20/155)	56% (87/155)	79% (118/150)
Average rank ratio of target gene	31.23%	12.93%	7.39%

**C. Congenital Heart Disease (CHD) test case**			

	**SUSPECTS**	**ENDEAVOUR**	**ToppGene**

Percentage of top 10% ranked target genes	32% (9/28)	50% (14/28)	64% (18/28)
Percentage of top 5% ranked target genes	18% (5/28)	14%(4/28)	25% (7/28)
Average rank ratio	25.03%	17.29%	17.35%

**D. Diabetic Retinopathy (DR) test case**			

	**SUSPECTS**	**ENDEAVOUR**	**ToppGene**

Percentage of top 10% ranked target genes	63% (17/27)	56% (15/27)	70% (19/27)
Percentage of top 5% ranked target genes	44% (12/27)	44% (12/27)	63% (17/27)
Average rank ratio	17.04%	13.31%	8.60%

Second, we studied the ROC curves based on *p-value *based scores. ENDEAVOUR provides ranking of the "target" gene based on *p-values *from order statistics, which are local *p-values*. In contrast, ToppGene provides *p-values *based on random sampling of the whole genome. ToppGene *p-value *based scores are therefore global measures of the similarity of the test genes to the training genes. As a result, sensitivity and specificity can also be defined based on the *p-value *based scores; specifically, sensitivity is the true positive rate (the proportion of detected "target" genes among all "target" genes) at a cutoff score, and specificity is the true negative rate (the proportion of "rejected" genes among all "non-target" genes) at the same cut-off level. For example, a sensitivity/specificity of 70/90 indicates that 70% of the "target" genes and 10% of the "non-target" genes have scores higher than a particular cut-off value.

### Evaluation of features used for gene prioritization in ToppGene

**Figure 2:**
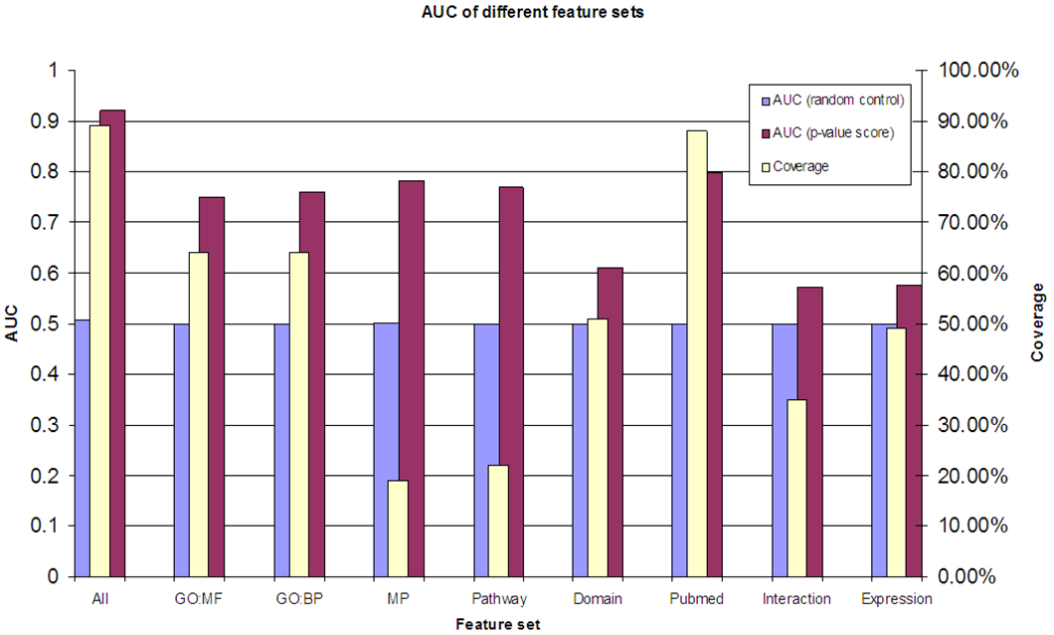
AUC of different feature sets. Red bars indicate the AUC scores based on each feature set, and blue bars are the corresponding random controls. Yellow bars indicate the coverage of each feature set in the whole genome. For example, mouse phenotype (MP) has AUC score 0.78 and covers 19% of genes in the whole genome. For each feature set, the ROC curve was generated using genes with annotations only.

To study the efficiency of different features (GO-Gene Ontology, MP-Mouse Phenotype, Pathways, PubMed, Protein Domains, Gene Expression and Protein Interactions), ROC curve of each of the feature sets was generated. Figure 2 shows the corresponding AUC scores of the ROC curves, depicting the relative performance of each feature set in the prioritization method. The mouse phenotype and PubMed showed the best performance while protein interactions and gene expression features performed poorly. In terms of coverage (the percentage of genes annotated with each of these features in the whole genome), PubMed was the best while MP had least coverage (only about 19% of known genes have at least one MP term association).

**Figure 3:**
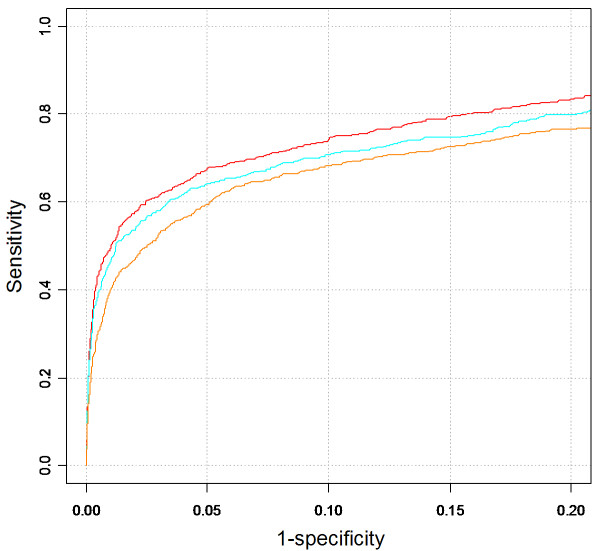
ROC curves of random-gene cross-validation based on scores. The red curve was generated using all features sets (AUC score 0.913). The blue curve was generated without Mouse Phenotype annotations (AUC score 0.893). The orange curve was generated without Mouse Phenotype and Pubmed annotations (AUC score 0.888). See text for the definitions of sensitivity and specificity.

To understand better the relative performance and the power of each of the features in gene prioritization, we tested ToppGene by performing cross-validations with one of the features left out. The performance decreased significantly only when MP was removed (see ROC curve in Figure 3). As expected, the best performance was recorded when all the features were considered for prioritization, with an AUC of 0.913 (see ROC curve in Figure 3) and a coverage of ~89%. For a cutoff score of 0.93, the sensitivity/specificity was 74/90. In other words, 74% of the "target" genes were included in the candidate list (about 9-fold reduction from the original test set).

### Comparison of ToppGene with SUSPECTS and PROSPECTR: Locus-region cross-validation

In this cross-validation we compared the performance of ToppGene with two other gene prioritization methods, namely, SUSPECTS [12] and PROSPECTR [8]. We used the same data set [6] that was used in SUSPECTS and PROSPECTR study (See Additional file 3 for a complete list of the data set). This data set contains a list of 29 OMIM diseases (each disease had at least known gene associations). For each cross-validation run, the training set was composed of all the genes related to a disease except the "target" gene. The test set was created by including all the genes in the 15 Mb *locus region *i.e. genes occurring in the 7.5 Mb flanking regions (5' and 3') of the "target" gene's chromosomal location along with the "target" gene itself.

PROSPECTR, which uses sequence features alone for gene prioritization, ranked the "target" gene in an average of top 31.23% in the prioritized test lists and among the top 5% about 20 times out of 155 (i.e. about 13%). On the other hand, SUSPECTS, which uses GO, protein domains, gene expression, and sequence features for gene prioritization, ranked the "target" genes in the top 5% of the prioritized lists 87 times out of 155 (~56%), and on average the "target" genes were ranked at top 12.93% in the prioritization results.

In comparison, ToppGene was able to rank the "target" gene among the top 5% of the prioritized lists for 118 times out of 150 (79%). Five genes in the original list were not present in the current NCBI Entrez Gene database and were therefore excluded. Thus, instead of 155 genes, 150 genes were used for this cross-validation test. On average, the "target" genes were ranked at top 7.39% in the prioritized lists using our approach (see section B of Table 3).

**Figure 4:**
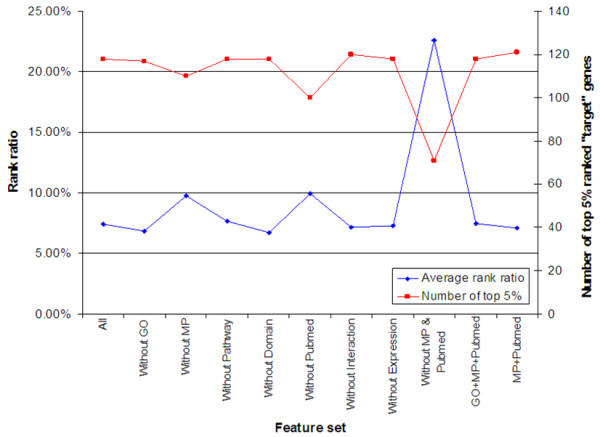
The performance of locus-region cross-validation using different feature sets. The average rank ratio (y-axis on the left) indicates the average rank ratio of the "target" genes in the resulting list, thus lower value corresponding to a better performance. At the same time, the higher the number of top 5% ranked "target" genes among total of 150 prioritizations (y-axis on the right), the better the performance. As a result, it's very clear that removing MP, PubMed or both resulted in significant drop of performance.

To evaluate the performance of the individual feature, we repeated the same locus-region cross-validation with one feature removed at a time (as described earlier under comparison of ToppGene with ENDEAVOUR). The performance did not change significantly if only GO, pathway, protein domains, protein interactions or gene expression features were excluded during gene prioritization. The performance however declined significantly when MP or PubMed was not included as one of the features in gene prioritization (see Table 4 and Figure 4).

**Table 4:** Performance summary of locus-region cross-validation using different feature sets. When either MP or PubMed, or both (MP + PubMed) were left out, the performance dropped significantly

**Features**	**Average rank ratio of "target" genes**	**Number of times "target" genes were ranked top 5%**	**Number of times "target" genes were ranked top 10%**
All	7.39%	118	125
GO + MP + PubMed	7.50%	118	126
MP + PubMed	7.08%	121	126
Without GO	6.84%	117	123
Without Pathway	7.66%	118	124
Without Domain	6.71%	118	124
Without Interaction	7.17%	120	124
Without Expression	7.28%	118	128
**Without MP**	**9.77%**	**110**	**117**
**Without Pubmed**	**9.91%**	**100**	**111**
**Without MP & Pubmed**	**22.61%**	**71**	**80**

### Comparison of ToppGene with ENDEAVOUR and SUSPECTS

#### Test case 1: Congenital heart disease (CHD)

We used 28 genes implicated in congenital heart disease (CHD) (see Additional file 4 for the complete list and comparison of relative rankings of "target" genes using different gene prioritization approaches) as the test case and prioritized the genes using the random-gene cross-validation method as described in the earlier sections. In each run, same training and test sets were submitted to SUSPECTS, ENDEAVOUR and ToppGene manually. Twenty-eight prioritizations were performed by each of the three methods and the average size of the test sets was 20 genes.

Following the prioritization, the "target" genes were ranked among the top 5% in the resulting lists 5, 4, and 7 times out of 28 (i.e., about 18%, 14%, and 25%), and in the top 10% 9, 14 and 18 times (about 32%, 50% and 64%) with SUSPECTS, ENDEAVOUR and ToppGene respectively. The average rank ratios of the "target" genes were 25.03%, 17.29% and 17.35% for SUSPECTS, ENDEAVOUR and our approach respectively (see section C of Table 3).

#### Test case 2: Diabetic retinopathy (DR)

A similar comparative analysis was repeated with diabetic retinopathy (DR) as a test case using locus-region cross-validation as described in previous section. The training set comprised 27 known genes implicated in DR (see Additional file 5 for the complete list and comparison of the relative rankings of the "target" genes using SUSPECTS, ENDEAVOUR and ToppGene) while the test sets comprised genes in the locus regions of the "target" genes.

The "target" genes were ranked among top 5% in the resulting lists 12 times out of 27 (~44%) with both SUSPECTS and ENDEAVOUR based gene prioritization. As witnessed in earlier comparisons, ToppGene again outperformed both SUSPECTS and ENDEAVOUR by ranking the "target" genes among top 5% in 17 times out of 27 (~63%). If we considered the top 10%, surprisingly SUSPECTS fared better than ENDEAVOUR and was close to ToppGene's performance. Thus, the "target" genes were ranked among the top 10% of the prioritized gene lists 17, 15 and 19 times (63%, 56% and 70%) respectively with SUSPECTS, ENDEAVOUR and ToppGene. The average rank ratios of the "target" genes were 17.04%, 13.31% and 8.49% for SUSPECTS, ENDEAVOUR and our approach respectively (see section D of Table 3).

### ToppGene implementation and access

The programs of our prioritization method are implemented purely in *JAVA*. Open source *JAVA *package FtpBean by Calvin Tai [19] is used to automatically download data and annotation files from Ftp servers. BioJava packages [20] are used to process UniProt records [21] and extract related protein domain information. GOLEM [22] source code was adapted and modified for dealing with ontology annotations. Colt [23] and Jakarta Commons-Math libraries [24] are used for statistical analysis. The fuzzy similarity measure and related functions are implemented locally.

Our prioritization method is available as a standalone web application [25]. The user interface is written in *JAVA *script, JSP and servlets, and integrated with the Tomcat web server. Users can enter the training and test sets of genes of interest as queries from the interface, and the application will display enriched themes (based on the GO, Pathways, Phenotype, Protein Domains, PubMed and Protein Interactions) in the training set genes along with annotated prioritized test genes. All the gene information and annotation data will be updated automatically except for pathways.

## Discussion

Traditionally there are two categories of approaches to compute the similarity between any two genes based on semantic annotations: pair-based and set-based [26]. In pair-based methods, an average or maximum of pairwise term information content is calculated as the similarity between the two genes. This will however cause inconsistency problems. Specifically, an average of pairwise term information content tends to underestimate the similarities (e.g. two identical genes have a similarity of less than 1) while a maximum of pairwise term information content tends to overestimate the similarity (e.g. two genes sharing one annotation term have similarity equal to 1). On the other hand, set-based similarity measures, such as Jaccard and Dice similarity [26], will generate 0 if the two genes do not share a common annotation term. This behavior is especially undesirable for annotation terms from ontologies. The fuzzy-based similarity measure adopted and applied in our approach can overcome these problems and therefore could generate a better similarity measure than the traditional methods.

Most of the current tools to enrich lists of genes or candidate gene prioritization are based on GO, gene expression or pathways [2,4,27,28]. Previous studies have also shown that integrating multiple lines of evidence is good for candidate gene analysis. However, to the best of our knowledge none of the previous candidate gene prioritization approaches used mouse phenotype features although the mouse is a key model organism for the analysis of mammalian developmental, physiological, and disease processes [14]. Additionally, there have been reports wherein a direct comparison of human and mouse phenotypes allowed for the rapid recognition of disease causal genes (for example, *ROR2 *as the Robinow syndrome gene [16]; the phenotype of the *Abcc6*-/- mouse shares calcification of elastic fibers with human Pseudoxanthoma elasticum, PXE, pathology, caused by mutations in human *ABCC6 *gene [15]). In this paper, for the first time, we use phenotype annotations for mouse orthologs of human genes as one line of evidence for candidate gene analysis. We are aware that comparing phenotypes between two different organisms may involve consideration of several issues. For instance, the mouse genotype may involve mutations to orthologs of one or more of the genes associated with a phenotype, but the mouse phenotype may not resemble the disease in human. Nevertheless, finding, for instance that targeted disruption of the mouse ortholog of human *CFC1 *gene (associated with visceral heterotaxy which is characterized by congenital anomalies that include complex cardiac malformations and *situs inversus *or *situs ambiguous *[29]) results in L-R laterality defects including cardiac malformations [30] can lead to novel and interesting hypotheses. Although, our results have conclusively demonstrated the utility of mouse phenotype data in human candidate gene analysis, there are some inherent limitations in using mouse phenotype annotations. For instance, MP is not a disease-centric ontology and the phenotype of a same gene mutation can vary depending on specific mouse strains or their genetic backgrounds. Most importantly, orthologous genes need not necessarily result in orthologous phenotypes. We are currently working on a more efficient cross-species phenome extrapolation where in the mouse phenotype terms are mapped to human phenotype concepts (from UMLS [31]) semantically ("orthologous phenotype") and the resultant orthologous genes associated with an orthologous phenotype are identified. How to efficiently utilize this kind of information in human disease candidate gene prioritization is a topic of future research.

Apart from the contribution of MP, improved performance of ToppGene over other methods can be attributed partially to the usage of more comprehensive data resources. For instance, unlike ENDEAVOUR, the pathway data set in ToppGene is not limited to KEGG resource. We compiled more than 700 additional pathways (associated with about 4800 human genes) from various sources (see Methods) and used for gene prioritization.

Our approach however has some limitations. First, by using a training set we assume that the disease genes we have yet to discover will be consistent with what is already known about a disease and/or its genetic basis which may not always be the case. Second, it is important to note that the annotations and analyses provided and the prioritization by our approach can only be as accurate as the underlying online sources from which the annotations are retrieved. Only one-fifth of the known human genes have pathway or phenotype annotations and there are still more than 40% genes whose functions are not defined (see Methods). Third, using an appropriate training set – although the difference was not significant, while cross-validating, we noted that using larger training sets (> 100 genes) would decrease the sensitivity and specificity of the prioritization when compared to using smaller training sets (7 to 21 genes).

## Conclusion

Existing disease candidate gene prioritization methodologies mine biological and functional information about candidate genes, and we believe that our system, ToppGene, can complement these existing approaches by using a novel method that mines mouse phenotype data. The aim of ToppGene is to generate likely candidates by extensive analysis of all known characteristics of genes, and is inevitably restricted by existing information be it GO annotation, pathways, phenotype or gene expression data. Through various examples, we demonstrate that ToppGene performs better than SUSPECTS, PROSEPCTR and ENDEAVOUR in candidate gene prioritization. However, it needs to be emphasized that our aim is not to prove that ToppGene prioritized genes are true disease genes but to aid in selection of a subset of most likely disease gene candidates from larger sets of disease-implicated genes identified by high throughput genome-wide techniques like linkage analysis and microarray analysis. For the first time, we have used the mouse phenotype data in human disease candidate gene analysis. Our results demonstrate that employing the mouse phenotype data improves candidate gene prioritization significantly and can therefore aid in the process of focusing the search for the most likely human disease gene candidates. Lastly, as the functional annotations of human and mouse genes improve, especially the mouse phenotype annotations, we envisage a proportional increase in the performance of ToppGene and strongly believe that it will be a valuable adjunct to wet lab experiments in human genetics and disease research.

## Methods

### Data sources

**Figure 5:**
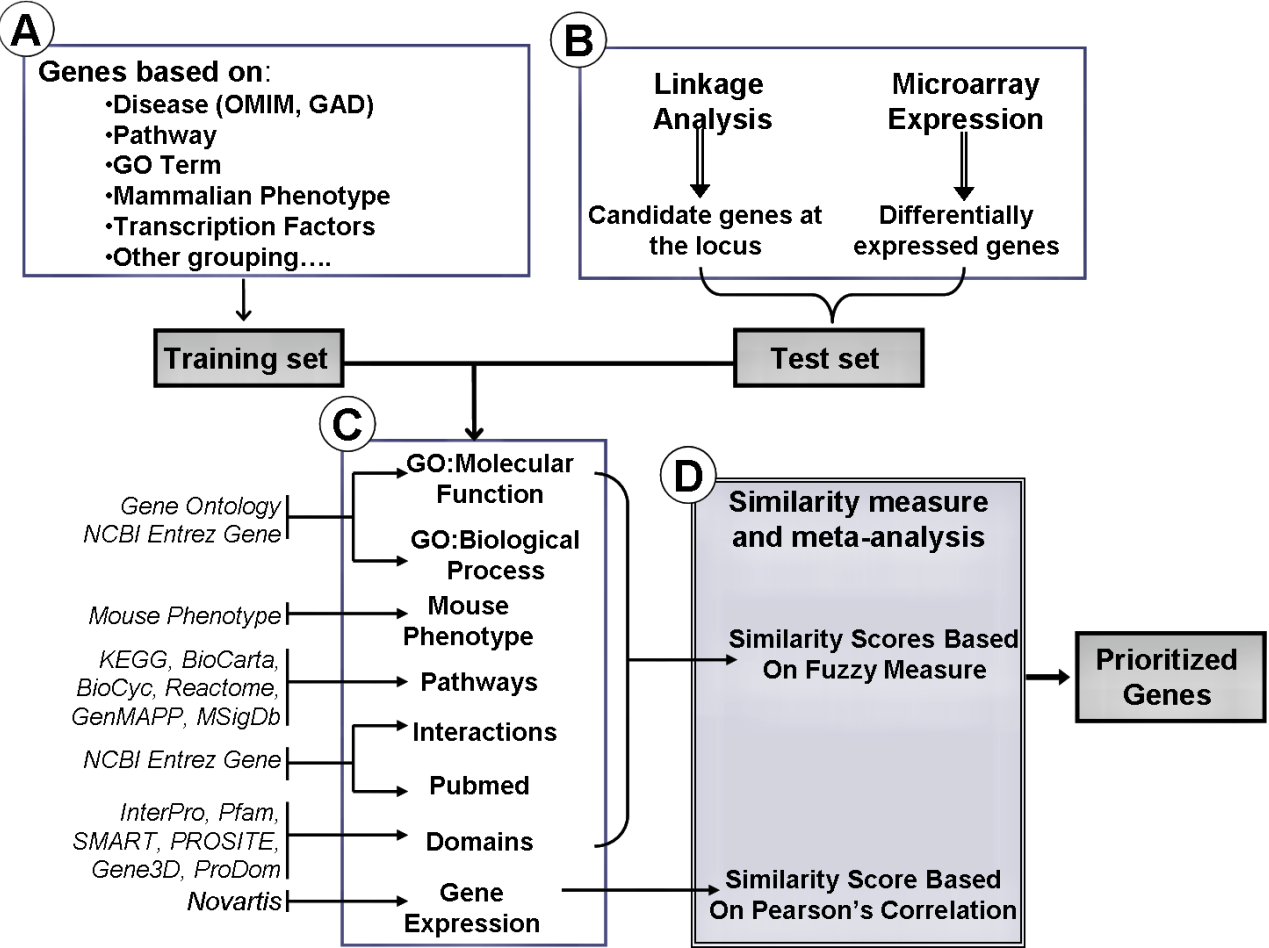
Schematic representation of gene prioritization. (A) Genes in the training set are selected based on their attributes or current gene annotations (genes associated with a disease, phenotype, pathway or a GO term). (B) Test gene source can be candidate genes from linkage analysis studies or genes differentially expressed in a particular disease or phenotype. (C) Enriched terms of the eight gene annotations, namely, GO: Molecular Function, GO: Biological Process, Mouse Phenotype, Pathways, Protein Interactions, Protein Domains and Gene Expression, compiled from various data sources, are obtained for the training set of genes. (D) A similarity score is generated for each annotation of each test gene by comparing to the enriched terms in the training set of genes. The final prioritized gene list is then computed based on the aggregated values of the eight similarity scores.

We used seven data sources (6 human-related and 1 mouse-related) to prioritize the gene candidates (see Figure 5).

1. Gene Ontology (GO): Gene Ontology [32] was downloaded from GO web site [33]. Corresponding human GO-gene annotations were downloaded from NCBI Entrez Gene ftp site [18]. This data set contained 15,068 human genes annotated with 7,124 unique GO terms. GO Molecular Function (GO:MF) and GO Biological Process (GO:BP) were considered as separate features since although they belong to the same annotation family (GO), they have separate roots and term spaces.

2. Mammalian Phenotype (MP): MP ontology [17], mouse gene phenotype annotations, and the corresponding orthologous genes from human were downloaded from Mouse Genome Informatics (MGI) website [34]. This data set contained 4329 human genes compiled by extrapolating the mouse genes annotated with 4280 mouse phenotype terms.

3. Pathway: Gene-pathway annotations were compiled by combining data from KEGG [35], BioCarta [36], BioCyc [37], Reactome [38], GenMAPP [39], and MSigDb [40,3]. 4,860 human genes had at least one pathway association (a total of 780 pathways).

4. Protein Domains: Domain information of all gene products was collected by parsing the UniProt human records. This compiled gene-domain annotation data set contains 12,454 distinct genes annotated by 10,223 distinct domains from 6 protein domain databases: InterPro [41], Pfam [42], SMART [43], PROSITE [44], Gene3D [45] and ProDom [46].

5. PubMed: Gene-PubMed ID relations were downloaded from NCBI Entrez Gene ftp site [18]. This data set contained 25,294 distinct genes associated with at least one PMID (a total of more than 142,000 PubMed abstract). About 32% (44,806) of these papers were associated with at least two genes.

6. Protein Interactions: The gene product interaction complex relations were downloaded from NCBI Entrez Gene ftp site [18]. This data set contained 8,040 distinct genes from 19,714 distinct interaction complexes from 3 interaction databases: HPRD [47], BIND [48], and BioGRID [49].

7. Gene Expression: Human microarray expression data (Series GSE1133) from Genomics Institute of the Novartis Research Foundation was obtained from the NCBI Gene Expression Omnibus (GEO) [50]. This dataset [51] contained expression values of 11,883 genes from 79 tissues from normal adult human body. Microarray expression CEL files were pre-processed using RMA algorithm. The annotations were created with a custom chip description file Hs133A_Hs_REFSEQ_8.cdf [52] to account for recent advances in human genomics, followed by per gene median normalization. Each gene was represented by a vector of size 79, corresponding to the expression values of the 79 normal adult human tissues.

### Pre-processing of annotation terms

A pre-processing step was performed prior to using the eight features for candidate gene prioritization. The information content values of all categorical annotation terms, namely, GO:MF, GO:BP, MP, Pathways, Protein Domains, PubMed, and Protein Interaction annotations, were calculated. The information content (g^i^) of annotation term T_i _of a gene was defined in the following way:

gi=−ln(p(Ti))max{−ln(p(Tj))}︸all Tj in the taxonomy,
 MathType@MTEF@5@5@+=feaafiart1ev1aaatCvAUfKttLearuWrP9MDH5MBPbIqV92AaeXatLxBI9gBaebbnrfifHhDYfgasaacH8akY=wiFfYdH8Gipec8Eeeu0xXdbba9frFj0=OqFfea0dXdd9vqai=hGuQ8kuc9pgc9s8qqaq=dirpe0xb9q8qiLsFr0=vr0=vr0dc8meaabaqaciaacaGaaeqabaqabeGadaaakeaacqqGNbWzdaahaaWcbeqaaiabbMgaPbaakiabg2da9maalaaabaaccaGae8NeI0IaeeiBaWMaeeOBa4MaeeikaGIaeeiCaaNaeeikaGIaeeivaq1aaSbaaSqaaiabbMgaPbqabaGccqGGPaqkcqGGPaqkaeaacqqGTbqBcqqGHbqycqqG4baEdaagaaqaaiabbUha7jab=jHiTiabbYgaSjabb6gaUjabbIcaOiabbchaWjabbIcaOiabbsfaunaaBaaaleaacqqGQbGAaeqaaOGaeiykaKIaeiykaKIaeiyFa0haleaacqqGHbqycqqGSbaBcqqGSbaBcqqGGaaicqqGubavdaWgaaadbaGaeeOAaOgabeaaliabbccaGiabbMgaPjabb6gaUjabbccaGiabbsha0jabbIgaOjabbwgaLjabbccaGiabbsha0jabbggaHjabbIha4jabb+gaVjabb6gaUjabb+gaVjabb2gaTjabbMha5bGccaGL44paaaGaeiilaWcaaa@6CFF@

where

p(Ti)=count(occurrence of Ti and children of Ti in case of ontological annotation)count(occurrence of all terms in the same annotation set)
 MathType@MTEF@5@5@+=feaafiart1ev1aaatCvAUfKttLearuWrP9MDH5MBPbIqV92AaeXatLxBI9gBaebbnrfifHhDYfgasaacH8akY=wiFfYdH8Gipec8Eeeu0xXdbba9frFj0=OqFfea0dXdd9vqai=hGuQ8kuc9pgc9s8qqaq=dirpe0xb9q8qiLsFr0=vr0=vr0dc8meaabaqaciaacaGaaeqabaqabeGadaaakeaacqWGWbaCcqGGOaakcqWGubavdaWgaaWcbaGaemyAaKgabeaakiabcMcaPiabg2da9maalaaabaGaee4yamMaee4Ba8MaeeyDauNaeeOBa4MaeeiDaqNaeeikaGIaee4Ba8Maee4yamMaee4yamMaeeyDauNaeeOCaiNaeeOCaiNaeeyzauMaeeOBa4Maee4yamMaeeyzauMaeeiiaaIaee4Ba8MaeeOzayMaeeiiaaIaeeivaq1aaSbaaSqaaiabbMgaPbqabaGccqqGGaaicqqGHbqycqqGUbGBcqqGKbazcqqGGaaicqqGJbWycqqGObaAcqqGPbqAcqqGSbaBcqqGKbazcqqGYbGCcqqGLbqzcqqGUbGBcqqGGaaicqqGVbWBcqqGMbGzcqqGGaaicqqGubavdaWgaaWcbaGaeeyAaKgabeaakiabbccaGiabbMgaPjabb6gaUjabbccaGiabbogaJjabbggaHjabbohaZjabbwgaLjabbccaGiabb+gaVjabbAgaMjabbccaGiabb+gaVjabb6gaUjabbsha0jabb+gaVjabbYgaSjabb+gaVjabbEgaNjabbMgaPjabbogaJjabbggaHjabbYgaSjabbccaGiabbggaHjabb6gaUjabb6gaUjabb+gaVjabbsha0jabbggaHjabbsha0jabbMgaPjabb+gaVjabb6gaUjabcMcaPaqaaiabbogaJjabb+gaVjabbwha1jabb6gaUjabbsha0jabbIcaOiabb+gaVjabbogaJjabbogaJjabbwha1jabbkhaYjabbkhaYjabbwgaLjabb6gaUjabbogaJjabbwgaLjabbccaGiabb+gaVjabbAgaMjabbccaGiabbggaHjabbYgaSjabbYgaSjabbccaGiabbsha0jabbwgaLjabbkhaYjabb2gaTjabbohaZjabbccaGiabbMgaPjabb6gaUjabbccaGiabbsha0jabbIgaOjabbwgaLjabbccaGiabbohaZjabbggaHjabb2gaTjabbwgaLjabbccaGiabbggaHjabb6gaUjabb6gaUjabb+gaVjabbsha0jabbggaHjabbsha0jabbMgaPjabb+gaVjabb6gaUjabbccaGiabbohaZjabbwgaLjabbsha0jabbMcaPaaaaaa@DBF3@

### Processing of training set genes

The training process was to create a representative profile of the training genes based on all the 8 annotations (features). For categorical gene annotations this process was to identify the over-representative terms from the training genes. Hypergeometric distribution with Bonferroni correction was used as the standard method. For numeric gene annotation, i.e. microarray expression levels, the training process generated the average (a vector of size 79) of all the training genes.

#### Similarity measure

Again different methods were used for similarity measures of categorical and numeric annotations. Fuzzy measure-based similarity measure was applied for categorical terms. The following part explains the method in detail.

If G = {T_1_,..., T_n_} denotes the set of annotation terms of a gene, a Sugeno fuzzy measure, g, is a real valued function g: 2^G ^→ [0, 1], satisfying

1) *g*(Φ) = 0 *and g*(*G*) = 1,

2) *g*(*A*) ≤ *g*(*B*) *if A *⊆ *B*, and

3) For all *A*, *B *⊆ *G *with *A *⋂ *B *= Φ,

*g*(*A *∪ *B *= *g*(*A*) + *g*(*B*) + *λg*(*A*)*g*(*B*) for some *λ * > -1.

For a given gene annotation set G, the parameter *λ *of its Sugeno fuzzy measure can be determined uniquely by solving the following equation:

(1+λ)=∏i=1n(1+λgi)for λ>−1.
 MathType@MTEF@5@5@+=feaafiart1ev1aaatCvAUfKttLearuWrP9MDH5MBPbIqV92AaeXatLxBI9gBaebbnrfifHhDYfgasaacH8akY=wiFfYdH8Gipec8Eeeu0xXdbba9frFj0=OqFfea0dXdd9vqai=hGuQ8kuc9pgc9s8qqaq=dirpe0xb9q8qiLsFr0=vr0=vr0dc8meaabaqaciaacaGaaeqabaqabeGadaaakeaafaqabeqacaaabaGaeiikaGIaeGymaeJaey4kaSccciGae83UdWMaeiykaKIaeyypa0ZaaebCaeaacqGGOaakcqaIXaqmcqGHRaWkcqWF7oaBcqWGNbWzdaahaaWcbeqaaiabdMgaPbaakiabcMcaPaWcbaGaemyAaKMaeyypa0JaeGymaedabaGaemOBa4ganiabg+GivdaakeaacqqGMbGzcqqGVbWBcqqGYbGCcqqGGaaicqWF7oaBcqGH8aapcqGHsislcqaIXaqmcqGGUaGlaaaaaa@4C69@

where g^i ^is the fuzzy density of term T_i_, or the information content obtained in the pre-processing step, and n is the number of terms in G.

Fuzzy measure-based similarity (FMS) of two sets G_1 _and G_2 _of annotation terms is defined as

SFMS(G1, G2) = g1(G1∩G2)+g2(G1∩G2)2,
 MathType@MTEF@5@5@+=feaafiart1ev1aaatCvAUfKttLearuWrP9MDH5MBPbIqV92AaeXatLxBI9gBaebbnrfifHhDYfgasaacH8akY=wiFfYdH8Gipec8Eeeu0xXdbba9frFj0=OqFfea0dXdd9vqai=hGuQ8kuc9pgc9s8qqaq=dirpe0xb9q8qiLsFr0=vr0=vr0dc8meaabaqaciaacaGaaeqabaqabeGadaaakeaacqqGtbWudaWgaaWcbaGaeeOrayKaeeyta0Kaee4uamfabeaakiabbIcaOiabbEeahnaaBaaaleaacqqGXaqmaeqaaOGaeeilaWIaeeiiaaIaee4raC0aaSbaaSqaaiabbkdaYaqabaGccqqGPaqkcqqGGaaicqGH9aqpcqqGGaaidaWcaaqaaiabbEgaNnaaBaaaleaacqqGXaqmaeqaaOGaeiikaGIaem4raC0aaSbaaSqaaiabigdaXaqabaGccqGHPiYXcqWGhbWrdaWgaaWcbaGaeGOmaidabeaakiabcMcaPiabgUcaRiabbEgaNnaaBaaaleaacqqGYaGmaeqaaOGaeiikaGIaem4raC0aaSbaaSqaaiabigdaXaqabaGccqGHPiYXcqWGhbWrdaWgaaWcbaGaeGOmaidabeaakiabcMcaPaqaaiabbkdaYaaacqGGSaalaaa@5303@

which can be derived based on the values of *λ*_1 _and *λ*_2 _determined using equation (3). For ontological terms, the augmented FMS (AFMS) was used to account for the hierarchical structure of ontology annotations.

SAFMS(G1,G2)=g1+([G1∩G2]+)+g2+([G1∩G2]+)2,
 MathType@MTEF@5@5@+=feaafiart1ev1aaatCvAUfKttLearuWrP9MDH5MBPbIqV92AaeXatLxBI9gBaebbnrfifHhDYfgasaacH8akY=wiFfYdH8Gipec8Eeeu0xXdbba9frFj0=OqFfea0dXdd9vqai=hGuQ8kuc9pgc9s8qqaq=dirpe0xb9q8qiLsFr0=vr0=vr0dc8meaabaqaciaacaGaaeqabaqabeGadaaakeaacqWGtbWudaWgaaWcbaGaemyqaeKaemOrayKaemyta0Kaem4uamfabeaakiabcIcaOiabdEeahnaaBaaaleaacqaIXaqmaeqaaOGaeiilaWIaem4raC0aaSbaaSqaaiabikdaYaqabaGccqGGPaqkcqGH9aqpdaWcaaqaaiabdEgaNnaaDaaaleaacqaIXaqmaeaacqGHRaWkaaGccqGGOaakcqGGBbWwcqWGhbWrdaWgaaWcbaGaeGymaedabeaakiabgMIihlabdEeahnaaBaaaleaacqaIYaGmaeqaaOGaeiyxa01aaWbaaSqabeaacqGHRaWkaaGccqGGPaqkcqGHRaWkcqWGNbWzdaqhaaWcbaGaeGOmaidabaGaey4kaScaaOGaeiikaGIaei4waSLaem4raC0aaSbaaSqaaiabigdaXaqabaGccqGHPiYXcqWGhbWrdaWgaaWcbaGaeGOmaidabeaakiabc2faDnaaCaaaleqabaGaey4kaScaaOGaeiykaKcabaGaeGOmaidaaiabcYcaSaaa@5AE7@

where [G1 ∩ G_2_]^+ ^= [G_1_^+ ^∩ G_2_^+^] = [G_1 _∩ G_2_] ∪ {T_1i_, T_2j_}, G_1_^+ ^= G_1 _∪ {T_1i_, T_2j_}, G_2_^+ ^= G_2 _∪ {T_1i_, T_2j_}, and {T_1i_, T_2j_} denotes the set of most specific common ancestors of every pair of terms (T_1i_, T_2j_) from G_1 _and G_2_. This ensures for two genes annotated by ontological terms, even though they don't share common terms, the similarity measure is > 0 (See Popescu et al [26] for additional details). For numeric annotation, i.e. the microarray expression values, the similarity score was calculated as the Pearson correlation of the two expression vectors of the two genes.

### Processing of the test set genes

In this step, each of the genes from the test set was compared to the representative profile of the training set. As described earlier, the training profile contained the overrepresented terms from the training genes for all categorical annotations and the average vector for the expression values. For a test gene, a similarity score to the training profile for each of the eight features was derived using the methods mentioned in the previous section. The test gene was then summarized by the 8 similarity scores. In case of missing value (for instance, lack of one or more annotations for a test gene), the score was set to -1. Otherwise, it is a real value in [0, 1].

In order to combine the 8 similarity scores into an overall score, we applied a statistical meta-analysis. A *p-value *of each annotation of a test gene G was derived by random sampling from the whole genome. The *p-value *of similarity score S_i _was defined as:

p(Si)=count of genes having score higher than G in the random samplecount of genes in the random sample containing annotation.
 MathType@MTEF@5@5@+=feaafiart1ev1aaatCvAUfKttLearuWrP9MDH5MBPbIqV92AaeXatLxBI9gBaebbnrfifHhDYfgasaacH8akY=wiFfYdH8Gipec8Eeeu0xXdbba9frFj0=OqFfea0dXdd9vqai=hGuQ8kuc9pgc9s8qqaq=dirpe0xb9q8qiLsFr0=vr0=vr0dc8meaabaqaciaacaGaaeqabaqabeGadaaakeaacqWGWbaCcqGGOaakcqWGtbWudaWgaaWcbaGaemyAaKgabeaakiabcMcaPiabg2da9maalaaabaGaee4yamMaee4Ba8MaeeyDauNaeeOBa4MaeeiDaqNaeeiiaaIaee4Ba8MaeeOzayMaeeiiaaIaee4zaCMaeeyzauMaeeOBa4MaeeyzauMaee4CamNaeeiiaaIaeeiAaGMaeeyyaeMaeeODayNaeeyAaKMaeeOBa4Maee4zaCMaeeiiaaIaee4CamNaee4yamMaee4Ba8MaeeOCaiNaeeyzauMaeeiiaaIaeeiAaGMaeeyAaKMaee4zaCMaeeiAaGMaeeyzauMaeeOCaiNaeeiiaaIaeeiDaqNaeeiAaGMaeeyyaeMaeeOBa4MaeeiiaaIaee4raCKaeeiiaaIaeeyAaKMaeeOBa4MaeeiiaaIaeeiDaqNaeeiAaGMaeeyzauMaeeiiaaIaeeOCaiNaeeyyaeMaeeOBa4MaeeizaqMaee4Ba8MaeeyBa0MaeeiiaaIaee4CamNaeeyyaeMaeeyBa0MaeeiCaaNaeeiBaWMaeeyzaugabaGaee4yamMaee4Ba8MaeeyDauNaeeOBa4MaeeiDaqNaeeiiaaIaee4Ba8MaeeOzayMaeeiiaaIaee4zaCMaeeyzauMaeeOBa4MaeeyzauMaee4CamNaeeiiaaIaeeyAaKMaeeOBa4MaeeiiaaIaeeiDaqNaeeiAaGMaeeyzauMaeeiiaaIaeeOCaiNaeeyyaeMaeeOBa4MaeeizaqMaee4Ba8MaeeyBa0MaeeiiaaIaee4CamNaeeyyaeMaeeyBa0MaeeiCaaNaeeiBaWMaeeyzauMaeeiiaaIaee4yamMaee4Ba8MaeeOBa4MaeeiDaqNaeeyyaeMaeeyAaKMaeeOBa4MaeeyAaKMaeeOBa4Maee4zaCMaeeiiaaIaeeyyaeMaeeOBa4MaeeOBa4Maee4Ba8MaeeiDaqNaeeyyaeMaeeiDaqNaeeyAaKMaee4Ba8MaeeOBa4gaaiabc6caUaaa@CB7C@

Fisher's inverse chi-square method, which states that  (assuming p_i_'s come from independent tests), was then applied to combine the *p-values *from multiple annotations into an overall *p-value*. Since the *p-values *of GO:MF and GO:BP were highly correlated, a single *p-value *was generated by taking the *p-value *of the average of GO:MF and GO:BP scores in the random sample. A pairwise Pearson correlation test result of the *p-values *is shown in Additional file 6. The final similarity score of the test gene was then obtained by 1 minus the combined *p-value*. We used random sampling to estimate the *p-values *because the density functions of the similarity scores were not easy to estimate, and although this process increased the computation time, for a reasonably large random sample the *p-values *were fairly stable.

## Authors' contributions

JC, BA and AJ conceived the study design, which was coordinated by AJ. JC designed and implemented the gene prioritization algorithms and along with AJ participated in the analysis and interpretation of results. HX carried out the expression data analysis. JC and AJ drafted the manuscript. All the authors have read and approved the final manuscript.

## Supplementary Material

Additional file 1 Comparison of ToppGene with other prioritization approaches – Workflow. This figure shows the details of the comparisons we performed to evaluate our approach with respect to other similar gene prioritization approaches. Click here for file

Additional file 2 List and ranking of genes in the 19 disease training sets used for validation. This file has the list of genes in the 19 disease training sets (randomly derived from Genetic Association Database, GAD and Online Mendelian Inheritance in Man, OMIM) used for validation along with the ranking of the "target" genes in random cross-validation. Click here for file

Additional file 3 List and ranking of "target" genes in locus-region cross-validation using different feature sets. This file has the details of the ranking of the "target" genes in locus-region cross-validation using different gene feature sets. When MP or PubMed annotations were excluded in the prioritization, the prioritization performance dropped significantly. Click here for file

Additional file 4 Comparison of relative rankings of "target" genes of congenital heart disease using SUSPECTS, ENDEAVOUR and ToppGene. The data provided represent the ranking results of "target" genes of congenital heart disease using SUSPECTS, ENDEAVOUR and ToppGene applications. Click here for file

Additional file 5 Comparison of relative rankings of "target" genes of diabetic retinopathy using SUSPECTS, ENDEAVOUR and ToppGene. The data provided represent the ranking results of "target" genes of diabetic retinopathy using SUSPECTS, ENDEAVOUR and ToppGene applications. Click here for file

Additional file 6 Pairwise Pearson correlation test result of the *p-values *of all the 7 features used for candidate gene prioritization. This figure shows the pairwise Pearson correlation test result of the *p-values *of all the features used for candidate gene prioritization. Click here for file

## References

[B1] Giallourakis C, Henson C, Reich M, Xie X, Mootha VK (2005). Disease gene discovery through integrative genomics. Annu Rev Genomics Hum Genet.

[B2] Dennis G, Sherman BT, Hosack DA, Yang J, Gao W, Lane HC, Lempicki RA (2003). DAVID: Database for Annotation, Visualization, and Integrated Discovery. Genome Biol.

[B3] Subramanian A, Tamayo P, Mootha VK, Mukherjee S, Ebert BL, Gillette MA, Paulovich A, Pomeroy SL, Golub TR, Lander ES, Mesirov JP (2005). Gene set enrichment analysis: a knowledge-based approach for interpreting genome-wide expression profiles. Proc Natl Acad Sci U S A.

[B4] Al-Shahrour F, Diaz-Uriarte R, Dopazo J (2004). FatiGO: a web tool for finding significant associations of Gene Ontology terms with groups of genes. Bioinformatics.

[B5] Freudenberg J, Propping P (2002). A similarity-based method for genome-wide prediction of disease-relevant human genes. Bioinformatics.

[B6] Turner FS, Clutterbuck DR, Semple CA (2003). POCUS: mining genomic sequence annotation to predict disease genes. Genome Biol.

[B7] Tiffin N, Kelso JF, Powell AR, Pan H, Bajic VB, Hide WA (2005). Integration of text- and data-mining using ontologies successfully selects disease gene candidates. Nucleic Acids Res.

[B8] Adie EA, Adams RR, Evans KL, Porteous DJ, Pickard BS (2005). Speeding disease gene discovery by sequence based candidate prioritization. BMC Bioinformatics.

[B9] Aerts S, Lambrechts D, Maity S, Van Loo P, Coessens B, De Smet F, Tranchevent LC, De Moor B, Marynen P, Hassan B, Carmeliet P, Moreau Y (2006). Gene prioritization through genomic data fusion. Nat Biotechnol.

[B10] Tiffin N, Adie E, Turner F, Brunner HG, van Driel MA, Oti M, Lopez-Bigas N, Ouzounis C, Perez-Iratxeta C, Andrade-Navarro MA, Adeyemo A, Patti ME, Semple CA, Hide W (2006). Computational disease gene identification: a concert of methods prioritizes type 2 diabetes and obesity candidate genes. Nucleic Acids Res.

[B11] Oti M, Brunner H (2007). The modular nature of genetic diseases. Clin Genet.

[B12] Adie EA, Adams RR, Evans KL, Porteous DJ, Pickard BS (2006). SUSPECTS: enabling fast and effective prioritization of positional candidates. Bioinformatics.

[B13] Mootha VK, Lepage P, Miller K, Bunkenborg J, Reich M, Hjerrild M, Delmonte T, Villeneuve A, Sladek R, Xu F, Mitchell GA, Morin C, Mann M, Hudson TJ, Robinson B, Rioux JD, Lander ES (2003). Identification of a gene causing human cytochrome c oxidase deficiency by integrative genomics. Proc Natl Acad Sci U S A.

[B14] Clarke AR (1994). Murine genetic models of human disease. Curr Opin Genet Dev.

[B15] Gorgels TG, Hu X, Scheffer GL, van der Wal AC, Toonstra J, de Jong PT, van Kuppevelt TH, Levelt CN, de Wolf A, Loves WJ, Scheper RJ, Peek R, Bergen AA (2005). Disruption of Abcc6 in the mouse: novel insight in the pathogenesis of pseudoxanthoma elasticum. Hum Mol Genet.

[B16] van Bokhoven H, Celli J, Kayserili H, van Beusekom E, Balci S, Brussel W, Skovby F, Kerr B, Percin EF, Akarsu N, Brunner HG (2000). Mutation of the gene encoding the ROR2 tyrosine kinase causes autosomal recessive Robinow syndrome. Nat Genet.

[B17] Smith CL, Goldsmith CA, Eppig JT (2005). The Mammalian Phenotype Ontology as a tool for annotating, analyzing and comparing phenotypic information. Genome Biol.

[B18] Entrez Gene. http://www.ncbi.nlm.nih.gov/entrez/query.fcgi?db=gene.

[B19] Tai C Open source JAVA package FtpBean. http://www.geocities.com/SiliconValley/Code/9129.

[B20] BioJava Package. http://biojava.org.

[B21] Peri S, Navarro JD, Amanchy R, Kristiansen TZ, Jonnalagadda CK, Surendranath V, Niranjan V, Muthusamy B, Gandhi TK, Gronborg M, Ibarrola N, Deshpande N, Shanker K, Shivashankar HN, Rashmi BP, Ramya MA, Zhao Z, Chandrika KN, Padma N, Harsha HC, Yatish AJ, Kavitha MP, Menezes M, Choudhury DR, Suresh S, Ghosh N, Saravana R, Chandran S, Krishna S, Joy M, Anand SK, Madavan V, Joseph A, Wong GW, Schiemann WP, Constantinescu SN, Huang L, Khosravi-Far R, Steen H, Tewari M, Ghaffari S, Blobe GC, Dang CV, Garcia JG, Pevsner J, Jensen ON, Roepstorff P, Deshpande KS, Chinnaiyan AM, Hamosh A, Chakravarti A, Pandey A (2003). Development of human protein reference database as an initial platform for approaching systems biology in humans. Genome Res.

[B22] GOLEM. http://function.princeton.edu/GOLEM/download.html.

[B23] Colt. http://dsd.lbl.gov/~hoschek/colt.

[B24] Jakarta Commons-Math libraries. http://jakarta.apache.org/commons/math.

[B25] ToppGene. http://toppgene.cchmc.org.

[B26] Popescu M, Keller JM, Mitchell JA (2006). Fuzzy Measures on the Gene Ontology for Gene Product Similarity. IEEE/ACM Trans Comput Biol Bioinform.

[B27] Khatri P, Bhavsar P, Bawa G, Draghici S (2004). Onto-Tools: an ensemble of web-accessible, ontology-based tools for the functional design and interpretation of high-throughput gene expression experiments. Nucleic Acids Res.

[B28] Masseroli M, Galati O, Pinciroli F (2005). GFINDer: genetic disease and phenotype location statistical analysis and mining of dynamically annotated gene lists. Nucleic Acids Res.

[B29] Bamford RN, Roessler E, Burdine RD, Saplakoglu U, dela Cruz J, Splitt M, Goodship JA, Towbin J, Bowers P, Ferrero GB, Marino B, Schier AF, Shen MM, Muenke M, Casey B (2000). Loss-of-function mutations in the EGF-CFC gene CFC1 are associated with human left-right laterality defects. Nat Genet.

[B30] Yan YT, Gritsman K, Ding J, Burdine RD, Corrales JD, Price SM, Talbot WS, Schier AF, Shen MM (1999). Conserved requirement for EGF-CFC genes in vertebrate left-right axis formation. Genes Dev.

[B31] Bodenreider O (2004). The Unified Medical Language System (UMLS): integrating biomedical terminology. Nucleic Acids Res.

[B32] Harris MA, Clark J, Ireland A, Lomax J, Ashburner M, Foulger R, Eilbeck K, Lewis S, Marshall B, Mungall C, Richter J, Rubin GM, Blake JA, Bult C, Dolan M, Drabkin H, Eppig JT, Hill DP, Ni L, Ringwald M, Balakrishnan R, Cherry JM, Christie KR, Costanzo MC, Dwight SS, Engel S, Fisk DG, Hirschman JE, Hong EL, Nash RS, Sethuraman A, Theesfeld CL, Botstein D, Dolinski K, Feierbach B, Berardini T, Mundodi S, Rhee SY, Apweiler R, Barrell D, Camon E, Dimmer E, Lee V, Chisholm R, Gaudet P, Kibbe W, Kishore R, Schwarz EM, Sternberg P, Gwinn M, Hannick L, Wortman J, Berriman M, Wood V, de la Cruz N, Tonellato P, Jaiswal P, Seigfried T, White R (2004). The Gene Ontology (GO) database and informatics resource. Nucleic Acids Res.

[B33] The Gene Ontology. http://www.geneontology.org/ontology/gene_ontology_edit.obo.

[B34] MGI Mouse Genome Informatics. http://www.informatics.jax.org/.

[B35] Kanehisa M, Goto S, Hattori M, Aoki-Kinoshita KF, Itoh M, Kawashima S, Katayama T, Araki M, Hirakawa M (2006). From genomics to chemical genomics: new developments in KEGG. Nucleic Acids Res.

[B36] Biocarta Pathways. http://biocarta.com.

[B37] Karp PD, Ouzounis CA, Moore-Kochlacs C, Goldovsky L, Kaipa P, Ahren D, Tsoka S, Darzentas N, Kunin V, Lopez-Bigas N (2005). Expansion of the BioCyc collection of pathway/genome databases to 160 genomes. Nucleic Acids Res.

[B38] Joshi-Tope G, Gillespie M, Vastrik I, D'Eustachio P, Schmidt E, de Bono B, Jassal B, Gopinath GR, Wu GR, Matthews L, Lewis S, Birney E, Stein L (2005). Reactome: a knowledgebase of biological pathways. Nucleic Acids Res.

[B39] Dahlquist KD, Salomonis N, Vranizan K, Lawlor SC, Conklin BR (2002). GenMAPP, a new tool for viewing and analyzing microarray data on biological pathways. Nat Genet.

[B40] MSigDB: Molecular Signature Database. http://www.broad.mit.edu/gsea/msigdb/msigdb_index.html.

[B41] Mulder NJ, Apweiler R, Attwood TK, Bairoch A, Bateman A, Binns D, Bork P, Buillard V, Cerutti L, Copley R, Courcelle E, Das U, Daugherty L, Dibley M, Finn R, Fleischmann W, Gough J, Haft D, Hulo N, Hunter S, Kahn D, Kanapin A, Kejariwal A, Labarga A, Langendijk-Genevaux PS, Lonsdale D, Lopez R, Letunic I, Madera M, Maslen J, McAnulla C, McDowall J, Mistry J, Mitchell A, Nikolskaya AN, Orchard S, Orengo C, Petryszak R, Selengut JD, Sigrist CJ, Thomas PD, Valentin F, Wilson D, Wu CH, Yeats C (2007). New developments in the InterPro database. Nucleic Acids Res.

[B42] Finn RD, Mistry J, Schuster-Bockler B, Griffiths-Jones S, Hollich V, Lassmann T, Moxon S, Marshall M, Khanna A, Durbin R, Eddy SR, Sonnhammer EL, Bateman A (2006). Pfam: clans, web tools and services. Nucleic Acids Res.

[B43] Letunic I, Copley RR, Pils B, Pinkert S, Schultz J, Bork P (2006). SMART 5: domains in the context of genomes and networks. Nucleic Acids Res.

[B44] Hulo N, Bairoch A, Bulliard V, Cerutti L, De Castro E, Langendijk-Genevaux PS, Pagni M, Sigrist CJ (2006). The PROSITE database. Nucleic Acids Res.

[B45] Yeats C, Maibaum M, Marsden R, Dibley M, Lee D, Addou S, Orengo CA (2006). Gene3D: modelling protein structure, function and evolution. Nucleic Acids Res.

[B46] Bru C, Courcelle E, Carrere S, Beausse Y, Dalmar S, Kahn D (2005). The ProDom database of protein domain families: more emphasis on 3D. Nucleic Acids Res.

[B47] Mishra GR, Suresh M, Kumaran K, Kannabiran N, Suresh S, Bala P, Shivakumar K, Anuradha N, Reddy R, Raghavan TM, Menon S, Hanumanthu G, Gupta M, Upendran S, Gupta S, Mahesh M, Jacob B, Mathew P, Chatterjee P, Arun KS, Sharma S, Chandrika KN, Deshpande N, Palvankar K, Raghavnath R, Krishnakanth R, Karathia H, Rekha B, Nayak R, Vishnupriya G, Kumar HG, Nagini M, Kumar GS, Jose R, Deepthi P, Mohan SS, Gandhi TK, Harsha HC, Deshpande KS, Sarker M, Prasad TS, Pandey A (2006). Human protein reference database--2006 update. Nucleic Acids Res.

[B48] Bader GD, Betel D, Hogue CW (2003). BIND: the Biomolecular Interaction Network Database. Nucleic Acids Res.

[B49] Stark C, Breitkreutz BJ, Reguly T, Boucher L, Breitkreutz A, Tyers M (2006). BioGRID: a general repository for interaction datasets. Nucleic Acids Res.

[B50] NCBI Gene Expression Omnibus (GEO). http://www.ncbi.nlm.nih.gov/projects/geo/.

[B51] Su AI, Wiltshire T, Batalov S, Lapp H, Ching KA, Block D, Zhang J, Soden R, Hayakawa M, Kreiman G, Cooke MP, Walker JR, Hogenesch JB (2004). A gene atlas of the mouse and human protein-encoding transcriptomes. Proc Natl Acad Sci U S A.

[B52] Dai M, Wang P, Boyd AD, Kostov G, Athey B, Jones EG, Bunney WE, Myers RM, Speed TP, Akil H, Watson SJ, Meng F (2005). Evolving gene/transcript definitions significantly alter the interpretation of GeneChip data. Nucleic Acids Res.

