# Supplementary material for: Improved human disease candidate gene prioritization using mouse phenotype
Source: BMC Bioinformatics. 2007 Oct 16;8:392. doi: 10.1186/1471-2105-8-392 (PMC2194797; doi:10.1186/1471-2105-8-392)
Supplement: Additional file 1 — Comparison of ToppGene with other prioritization approaches – Workflow. This figure shows the details of the comparisons we performed to evaluate our approach with respect to other similar gene prioritization approaches. [file 1471-2105-8-392-S1.doc]

**Additional file 1**: To evaluate the performance of our approach and also compare it with other similar gene prioritization approaches, we performed two types of comparisons: large-scale cross-validations and small-scale test cases. For large-scale cross-validations, we used the same or similar training sets as mentioned in the previous methods. Specifically we compared ToppGene’s performance with ENDEAVOUR using random-gene cross-validation; and with PROSPECTR and SUSPECTS, we used locus-region cross-validation. Further, as test cases, we selected two diseases, congenital heart defects (CHD) and diabetic retinopathy (DR), and compared the prioritization performance of ToppGene with SUSPECTS and ENDEAVOUR.

Comparison of ToppGene with other applications

Large scale cross-validation

Test cases

Random cross-validation

Locus-region cross-validation

Training: 19 diseases

Test: random genes

Compare with ENDEAVOUR

Training: 29 OMIM diseases

Test: locus region genes

Compare with SUSPECTS & PROSPECTR

Congenital heart disease (CHD)

Diabetic retinopathy (DR)

Training: 28 disease genes

Test: random genes

Compare with ENDEAVOUR & SUSPECTS

Training: 27 disease genes

Test: locus region genes

Compare with ENDEAVOUR & SUSPECTS
