# Supplementary material for: Improved human disease candidate gene prioritization using mouse phenotype
Source: BMC Bioinformatics. 2007 Oct 16;8:392. doi: 10.1186/1471-2105-8-392 (PMC2194797; doi:10.1186/1471-2105-8-392)
Supplement: Additional file 2 — List and ranking of genes in the 19 disease training sets used for validation. This file has the list of genes in the 19 disease training sets (randomly derived from Genetic Association Database, GAD and Online Mendelian Inheritance in Man, OMIM) used for validation along with the ranking of the "target" genes in random cross-validation. [file 1471-2105-8-392-S2.doc]

**Additional file 2:** List of genes in the 19 disease training sets (randomly derived from Genetic Association Database, GAD and Online Mendelian Inheritance in Man, OMIM) used for validation along with the ranking of the “target” genes in random cross-validation. The size of the test set is 100 for all rows.

| **Disease name** | **“Target” Gene symbol** | **Rank** | **Score** |
| --- | --- | --- | --- |
| **Ischaemic stroke** | *ACE* | 3 | 0.987738 |
|  | *ALOX5AP* | 22 | 0.604783 |
|  | *APOA1* | 3 | 0.913695 |
|  | *APOE* | 2 | 0.991067 |
|  | *CAPN10* | 21 | 0.741975 |
|  | *CCL2* | 5 | 0.870269 |
|  | *CD14* | 1 | 0.999774 |
|  | *CRP* | 3 | 0.983348 |
|  | *EPHX2* | 15 | 0.805371 |
|  | *F12* | 1 | 0.999975 |
|  | *F13A1* | 1 | 0.999079 |
|  | *F2* | 1 | 1 |
|  | *F5* | 1 | 0.999998 |
|  | *F7* | 1 | 0.999999 |
|  | *FGA* | 1 | 1 |
|  | *FGB* | 2 | 0.999998 |
|  | *GP1BA* | 2 | 0.9996 |
|  | *HLA-B* | 3 | 0.998844 |
|  | *HLA-DQB1* | 1 | 0.9996 |
|  | *HLA-DRA* | 1 | 0.988556 |
|  | *HLA-DRB1* | 1 | 0.999123 |
|  | *ICAM1* | 1 | 1 |
|  | *IL1B* | 1 | 0.999758 |
|  | *IL6* | 1 | 0.999638 |
|  | *ITGA2* | 2 | 0.999632 |
|  | *ITGA2B* | 1 | 0.99999 |
|  | *ITGA3* | 1 | 0.994163 |
|  | *ITGA4* | 1 | 0.998576 |
|  | *ITGB3* | 1 | 0.999958 |
|  | *MIF* | 4 | 0.944159 |
|  | *MMP3* | 1 | 0.997401 |
|  | *MTHFR* | 6 | 0.952549 |
|  | *NOS3* | 6 | 0.973789 |
|  | *NPR3* | 36 | 0.636104 |
|  | *PDE4D* | 8 | 0.766679 |
|  | *PLAT* | 1 | 0.999977 |
|  | *PON1* | 4 | 0.931171 |
|  | *PROZ* | 2 | 0.983488 |
|  | *SELE* | 7 | 0.954048 |
|  | *SERPINA1* | 1 | 0.999808 |
|  | *SERPINE1* | 1 | 1 |
|  | *SLC9A1* | 37 | 0.394603 |
|  | *TLR4* | 2 | 0.998719 |
|  | *TNF* | 1 | 0.999847 |
| **Endometriosis** | *AHR* | 1 | 0.999759 |
|  | *AHSG* | 42 | 0.266169 |
|  | *AR* | 1 | 0.999837 |
|  | *ARNT* | 2 | 0.998545 |
|  | *CCL5* | 3 | 0.982625 |
|  | *CCR2* | 6 | 0.974446 |
|  | *CCR5* | 11 | 0.921554 |
|  | *COMT* | 2 | 0.99309 |
|  | *CTLA4* | 1 | 0.99897 |
|  | *CYP17A1* | 1 | 0.999441 |
|  | *CYP19A1* | 1 | 0.999972 |
|  | *CYP1A1* | 1 | 0.999976 |
|  | *CYP1B1* | 1 | 0.999993 |
|  | *CYP2E1* | 1 | 0.999997 |
|  | *EPHX1* | 1 | 0.998186 |
|  | *ESR1* | 2 | 0.9999 |
|  | *ESR2* | 2 | 0.999808 |
|  | *FAS* | 1 | 0.999938 |
|  | *FASLG* | 1 | 0.99995 |
|  | *GALT* | 6 | 0.857495 |
|  | *GSTM1* | 1 | 0.998973 |
|  | *GSTT1* | 3 | 0.985356 |
|  | *HLA-A* | 1 | 0.999998 |
|  | *HLA-B* | 1 | 0.999999 |
|  | *HLA-C* | 1 | 0.999997 |
|  | *HLA-DPB1* | 1 | 1 |
|  | *HLA-DQA1* | 1 | 0.999962 |
|  | *HLA-DQB1* | 1 | 1 |
|  | *HLA-DRB1* | 1 | 0.999999 |
|  | *HSD17B1* | 50 | 0.205087 |
|  | *ICAM1* | 1 | 1 |
|  | *IFNG* | 1 | 0.999097 |
|  | *IL4* | 1 | 0.999654 |
|  | *IL6* | 1 | 0.999912 |
|  | *MMP1* | 5 | 0.959589 |
|  | *MMP3* | 2 | 0.99737 |
|  | *MPO* | 23 | 0.714214 |
|  | *NAT2* | 3 | 0.965102 |
|  | *NRIP1* | 24 | 0.511319 |
|  | *PGR* | 1 | 0.998925 |
|  | *TNF* | 1 | 0.999998 |
|  | *TNFRSF1B* | 6 | 0.962625 |
|  | *VEGFA* | 2 | 0.993646 |
| **Autism** | *ADA* | 47 | 0.333331 |
|  | *CENTG2* | 26 | 0.522807 |
|  | *CMYA3* | 76 | 0 |
|  | *DCX* | 16 | 0.755457 |
|  | *EN2* | 33 | 0.340799 |
|  | *FMR1* | 8 | 0.871554 |
|  | *FOXP2* | 22 | 0.655679 |
|  | *FRAXA* | 81 | 0 |
|  | *FRAXE* | 81 | 0 |
|  | *FRAXF* | 79 | 0 |
|  | *GABRA2* | 1 | 0.999997 |
|  | *GABRA4* | 1 | 0.999994 |
|  | *GABRA5* | 1 | 0.999995 |
|  | *GABRB3* | 1 | 0.999996 |
|  | *GABRG1* | 1 | 0.999999 |
|  | *GABRG3* | 1 | 0.999984 |
|  | *GLO1* | 30 | 0.454208 |
|  | *GRIK2* | 1 | 0.999805 |
|  | *GRM8* | 1 | 0.998573 |
|  | *GRPR* | 1 | 0.998537 |
|  | *HLA-DRB1* | 12 | 0.827365 |
|  | *HOXA1* | 27 | 0.395783 |
|  | *HRAS* | 9 | 0.867285 |
|  | *HTR2A* | 1 | 0.991093 |
|  | *MAOA* | 7 | 0.861708 |
|  | *MECP2* | 8 | 0.753152 |
|  | *MED12* | 20 | 0.581906 |
|  | *NF1* | 29 | 0.546843 |
|  | *NLGN3* | 14 | 0.575648 |
|  | *NLGN4X* | 8 | 0.685728 |
|  | *OMG* | 48 | 0.251777 |
|  | *OXTR* | 3 | 0.991981 |
|  | *PTEN* | 34 | 0.426848 |
|  | *PTPRZ1* | 3 | 0.93074 |
|  | *RELN* | 27 | 0.546475 |
|  | *SERPINE1* | 28 | 0.408427 |
|  | *SLC25A12* | 5 | 0.820942 |
|  | *SLC6A4* | 8 | 0.946161 |
|  | *TH* | 9 | 0.851 |
|  | *TPH2* | 40 | 0.531654 |
|  | *WNT2* | 25 | 0.437325 |
| **Lymphoma** | *ACE* | 11 | 0.98734 |
|  | *ADIPOQ* | 10 | 0.981485 |
|  | *BCL2* | 38 | 0.892718 |
|  | *BCL6* | 38 | 0.898567 |
|  | *CDKN1A* | 7 | 0.970588 |
|  | *COMT* | 2 | 0.998088 |
|  | *CTLA4* | 1 | 0.996883 |
|  | *CYP17A1* | 51 | 0.886086 |
|  | *CYP1A1* | 5 | 0.997593 |
|  | *CYP2E1* | 3 | 0.995405 |
|  | *EDN1* | 51 | 0.820776 |
|  | *EPHX1* | 1 | 0.999845 |
|  | *FCGR3A* | 1 | 0.999863 |
|  | *GSTM1* | 1 | 0.999752 |
|  | *GSTP1* | 4 | 0.99755 |
|  | *GSTT1* | 1 | 0.996275 |
|  | *HLA-A* | 1 | 0.999994 |
|  | *HLA-B* | 1 | 0.999998 |
|  | *HLA-C* | 1 | 1 |
|  | *HLA-DPB1* | 1 | 0.999998 |
|  | *HLA-DQA1* | 1 | 0.999999 |
|  | *HLA-DQB1* | 1 | 1 |
|  | *HLA-DRB1* | 1 | 1 |
|  | *IL1B* | 3 | 0.993411 |
|  | *LEP* | 5 | 0.99558 |
|  | *LEPR* | 2 | 0.999968 |
|  | *MTHFR* | 6 | 0.994935 |
|  | *MTR* | 6 | 0.987358 |
|  | *MYC* | 76 | 0 |
|  | *NAT1* | 6 | 0.987842 |
|  | *NAT2* | 1 | 0.99899 |
|  | *NQO1* | 13 | 0.959035 |
|  | *PRL* | 52 | 0.795326 |
|  | *RFC1* | 42 | 0.895242 |
|  | *SHMT1* | 3 | 0.999621 |
|  | *TLR4* | 18 | 0.955319 |
|  | *TNF* | 1 | 0.998572 |
|  | *TNFRSF1A* | 2 | 0.999937 |
|  | *TNFRSF1B* | 1 | 0.999727 |
|  | *TOP2A* | 45 | 0.829081 |
|  | *TP53* | 10 | 0.980114 |
|  | *TYMS* | 1 | 0.998115 |
| **Osteoarthritis** | *ADAMTS3* | 6 | 0.960099 |
|  | *ACAN* | 1 | 0.999896 |
|  | *AR* | 1 | 0.999982 |
|  | *ASPN* | 59 | 0.268371 |
|  | *BMP5* | 5 | 0.778581 |
|  | *CALM1* | 34 | 0.446199 |
|  | *COL11A1* | 2 | 0.994589 |
|  | *COL11A2* | 3 | 0.961169 |
|  | *COL1A1* | 1 | 0.999962 |
|  | *COL2A1* | 1 | 0.999989 |
|  | *COL9A1* | 1 | 0.999999 |
|  | *COL9A2* | 1 | 1 |
|  | *COL9A3* | 1 | 0.999992 |
|  | *COMP* | 3 | 0.997026 |
|  | *ENPP1* | 27 | 0.770536 |
|  | *ESR1* | 2 | 0.999048 |
|  | *ESR2* | 2 | 0.999115 |
|  | *FRZB* | 46 | 0.333075 |
|  | *HAPLN1* | 2 | 0.886494 |
|  | *HLA-DQA1* | 1 | 0.998172 |
|  | *HLA-DQB1* | 1 | 0.999982 |
|  | *HLA-DRB1* | 1 | 0.999509 |
|  | *IGF1* | 14 | 0.681234 |
|  | *IGFBP7* | 14 | 0.622812 |
|  | *IL1A* | 1 | 0.999804 |
|  | *IL1B* | 1 | 0.999993 |
|  | *IL1R1* | 1 | 0.999957 |
|  | *IL4R* | 2 | 0.994693 |
|  | *IL8* | 1 | 0.99995 |
|  | *LRP5* | 9 | 0.835557 |
|  | *MATN1* | 1 | 0.975804 |
|  | *MATN3* | 2 | 0.996861 |
|  | *MMP2* | 3 | 0.937519 |
|  | *PAPSS2* | 3 | 0.9559 |
|  | *SERPINA3* | 45 | 0.276936 |
|  | *SLC26A2* | 12 | 0.629475 |
|  | *TNF* | 1 | 0.999931 |
|  | *TNFAIP6* | 5 | 0.912459 |
|  | *TNFRSF1A* | 1 | 0.999999 |
|  | *TNFRSF1B* | 1 | 0.999966 |
|  | *VDR* | 1 | 0.999605 |
| **Myocardial ischemia** | *ABCA1* | 1 | 0.99999 |
|  | *ACE* | 3 | 0.993869 |
|  | *AGER* | 14 | 0.658473 |
|  | *AGT* | 1 | 0.997987 |
|  | *APOA1* | 1 | 0.999997 |
|  | *APOA5* | 1 | 0.999773 |
|  | *APOB* | 1 | 1 |
|  | *APOE* | 1 | 1 |
|  | *CETP* | 1 | 0.994665 |
|  | *F13A1* | 4 | 0.914883 |
|  | *F5* | 1 | 0.999996 |
|  | *F7* | 1 | 0.999122 |
|  | *FGB* | 1 | 0.999985 |
|  | *GP1BA* | 13 | 0.694769 |
|  | *HFE* | 56 | 0.320337 |
|  | *HIF1A* | 1 | 0.971672 |
|  | *HMOX1* | 2 | 0.974325 |
|  | *ITGA2* | 9 | 0.780451 |
|  | *ITGB3* | 9 | 0.834951 |
|  | *KCNMB1* | 19 | 0.588994 |
|  | *LPA* | 1 | 0.999973 |
|  | *LPL* | 1 | 0.999999 |
|  | *MMP3* | 1 | 0.987378 |
|  | *MTHFR* | 1 | 0.9786 |
|  | *NOS3* | 2 | 0.989877 |
|  | *P2RY12* | 28 | 0.404127 |
|  | *PECAM1* | 2 | 0.931016 |
|  | *PON1* | 1 | 0.99997 |
|  | *PON2* | 1 | 0.999955 |
|  | *PON3* | 1 | 0.999963 |
|  | *PPARA* | 3 | 0.983663 |
|  | *PPARG* | 8 | 0.711602 |
|  | *RAGE* | 32 | 0.40955 |
|  | *SELPLG* | 8 | 0.702734 |
|  | *SERPINA1* | 1 | 0.999883 |
|  | *SERPINE1* | 1 | 0.999996 |
|  | *THBS2* | 2 | 0.997893 |
|  | *THBS4* | 1 | 0.99617 |
|  | *TRIB3* | 31 | 0.522114 |
| **Neural tube defects** | *ALDH1A2* | 12 | 0.75008 |
|  | *APEX1* | 1 | 0.999701 |
|  | *BHMT* | 1 | 0.999987 |
|  | *BHMT2* | 1 | 0.999749 |
|  | *BMP4* | 1 | 0.935849 |
|  | *CBS* | 1 | 0.987898 |
|  | *CRABP1* | 24 | 0.471207 |
|  | *CRABP2* | 11 | 0.739492 |
|  | *CXCL6* | 36 | 0.340779 |
|  | *CYP26A1* | 3 | 0.951188 |
|  | *CYP26B1* | 1 | 0.995336 |
|  | *DPYD* | 13 | 0.716372 |
|  | *ERCC2* | 1 | 0.999827 |
|  | *FOLH1* | 6 | 0.834791 |
|  | *FOLR2* | 8 | 0.75384 |
|  | *MS* | 81 | 0 |
|  | *MTHFD1* | 1 | 0.974859 |
|  | *MTHFR* | 1 | 0.999994 |
|  | *MTR* | 1 | 1 |
|  | *MTRR* | 1 | 0.995981 |
|  | *MUT* | 3 | 0.974164 |
|  | *NOG* | 2 | 0.944813 |
|  | *OGG1* | 1 | 0.99461 |
|  | *PCMT1* | 2 | 0.975146 |
|  | *PDGFRA* | 5 | 0.80394 |
|  | *RFC1* | 1 | 0.97589 |
|  | *SHMT1* | 2 | 0.989314 |
|  | *SHMT2* | 1 | 0.998394 |
|  | *TCN1* | 2 | 0.97282 |
|  | *TCN2* | 2 | 0.932579 |
|  | *TERC* | 37 | 0.445811 |
|  | *TYMS* | 1 | 0.994934 |
|  | *UCP2* | 28 | 0.324174 |
|  | *XRCC1* | 1 | 0.99007 |
|  | *XRCC3* | 2 | 0.986676 |
|  | *ZIC1* | 1 | 0.979373 |
|  | *ZIC2* | 1 | 0.999489 |
|  | *ZIC3* | 1 | 0.999913 |
| **Cervical carcinoma** | *CCND1* | 33 | 0.970499 |
|  | *CCR2* | 4 | 0.996659 |
|  | *CDKN1A* | 1 | 0.99947 |
|  | *CYP2E1* | 50 | 0.922408 |
|  | *EPHX1* | 14 | 0.97678 |
|  | *FAS* | 1 | 0.999996 |
|  | *FASLG* | 1 | 0.999639 |
|  | *FHIT* | 25 | 0.980754 |
|  | *GSTM1* | 12 | 0.99051 |
|  | *HLA-A* | 1 | 0.999999 |
|  | *HLA-DPB1* | 1 | 1 |
|  | *HLA-DQA1* | 1 | 0.999999 |
|  | *HLA-DQB1* | 1 | 1 |
|  | *HLA-DRB1* | 1 | 0.999999 |
|  | *HRAS* | 6 | 0.989695 |
|  | *IFNA17* | 2 | 0.995073 |
|  | *IFNG* | 2 | 0.999183 |
|  | *IRF1* | 32 | 0.949377 |
|  | *KIR2DL1* | 1 | 1 |
|  | *KIR2DL2* | 1 | 1 |
|  | *KIR2DL3* | 1 | 0.999999 |
|  | *KIR2DL4* | 1 | 0.999999 |
|  | *KIR2DS4* | 1 | 0.999998 |
|  | *KIR3DL1* | 1 | 0.999991 |
|  | *KIR3DL2* | 1 | 0.999919 |
|  | *KIR3DL3* | 79 | 0 |
|  | *MICA* | 1 | 0.999333 |
|  | *MMP1* | 36 | 0.943393 |
|  | *NAT2* | 21 | 0.974799 |
|  | *OGG1* | 27 | 0.950615 |
|  | *SLC11A1* | 18 | 0.976823 |
|  | *ST14* | 31 | 0.970616 |
|  | *TAP1* | 4 | 0.998605 |
|  | *TCEAL1* | 13 | 0.980625 |
|  | *TNF* | 3 | 0.999997 |
|  | *TP53* | 1 | 0.999564 |
|  | *TP73* | 10 | 0.984285 |
|  | *XRCC1* | 33 | 0.953408 |
| **Epilepsy** | *ABCB1* | 2 | 0.984541 |
|  | *ATP1A2* | 2 | 0.968321 |
|  | *BDNF* | 4 | 0.931579 |
|  | *CACNA1A* | 1 | 0.996944 |
|  | *CACNA1H* | 4 | 0.883696 |
|  | *CHRNA4* | 1 | 0.999761 |
|  | *CSTB* | 27 | 0.572654 |
|  | *CYP2C19* | 2 | 0.980619 |
|  | *CYP2C9* | 1 | 0.998319 |
|  | *DBH* | 4 | 0.993146 |
|  | *DIDO1* | 22 | 0.682859 |
|  | *GABBR1* | 1 | 0.999945 |
|  | *GABRA5* | 1 | 1 |
|  | *GABRB1* | 1 | 0.99999 |
|  | *GABRB3* | 1 | 1 |
|  | *GABRD* | 1 | 1 |
|  | *GABRG2* | 1 | 1 |
|  | *GRIK1* | 1 | 0.998955 |
|  | *HLA-DRB1* | 12 | 0.681503 |
|  | *HP* | 26 | 0.413084 |
|  | *IL1B* | 36 | 0.38732 |
|  | *KCNJ10* | 1 | 0.999999 |
|  | *KCNJ3* | 1 | 0.999999 |
|  | *KCNJ6* | 1 | 1 |
|  | *KCNJ9* | 1 | 1 |
|  | *KCNQ2* | 1 | 0.972777 |
|  | *KCNQ3* | 3 | 0.980502 |
|  | *KHDRBS3* | 54 | 0.167316 |
|  | *LGI4* | 25 | 0.561556 |
|  | *MAOA* | 5 | 0.931594 |
|  | *OPRM1* | 3 | 0.998279 |
|  | *PAX6* | 36 | 0.374263 |
|  | *PDYN* | 6 | 0.836354 |
|  | *SCN1A* | 2 | 0.995126 |
|  | *SCN1B* | 3 | 0.989158 |
|  | *SLC4A3* | 7 | 0.87103 |
| **Grave's disease** | *C4A* | 26 | 0.455646 |
|  | *CD40* | 1 | 0.999977 |
|  | *CD40LG* | 1 | 0.999999 |
|  | *CTLA4* | 3 | 0.997301 |
|  | *ESR1* | 1 | 0.99966 |
|  | *ESR2* | 1 | 0.999941 |
|  | *FASLG* | 1 | 1 |
|  | *GC* | 28 | 0.458936 |
|  | *HLA-B* | 2 | 0.999999 |
|  | *HLA-DQA1* | 1 | 0.999969 |
|  | *HLA-DQB1* | 2 | 1 |
|  | *HLA-DRB1* | 2 | 0.99999 |
|  | *ICAM1* | 1 | 1 |
|  | *IFNG* | 1 | 1 |
|  | *IL12B* | 1 | 0.999999 |
|  | *IL13* | 1 | 1 |
|  | *IL1A* | 3 | 0.999919 |
|  | *IL1RN* | 1 | 0.999524 |
|  | *IL4* | 1 | 1 |
|  | *IL6* | 1 | 1 |
|  | *IL8* | 2 | 0.999881 |
|  | *INS* | 4 | 0.933408 |
|  | *IRF1* | 2 | 0.891391 |
|  | *LTA* | 1 | 1 |
|  | *PSMB9* | 4 | 0.897736 |
|  | *PTPN22* | 20 | 0.577653 |
|  | *SCGB3A2* | 30 | 0.389023 |
|  | *SLC26A4* | 3 | 0.947227 |
|  | *SUMO4* | 70 | 0.087339 |
|  | *TAP1* | 2 | 0.99485 |
|  | *TAP2* | 2 | 0.989149 |
|  | *TG* | 16 | 0.727091 |
|  | *THRB* | 3 | 0.994924 |
|  | *TNF* | 1 | 1 |
|  | *TSHR* | 2 | 0.991445 |
|  | *VDR* | 2 | 0.999506 |
| **Inflammatory bowel disease (IBD)** | *ABCB1* | 51 | 0.486439 |
|  | *ACE* | 5 | 0.994622 |
|  | *APC* | 23 | 0.966245 |
|  | *APOA1* | 31 | 0.937237 |
|  | *NOD2* | 1 | 0.999769 |
|  | *CD14* | 2 | 0.999443 |
|  | *CTLA4* | 2 | 0.998504 |
|  | *DLG5* | 31 | 0.939808 |
|  | *F13A1* | 12 | 0.984219 |
|  | *F13B* | 55 | 0.910559 |
|  | *F2* | 45 | 0.876143 |
|  | *HLA-DPA1* | 2 | 0.99974 |
|  | *HLA-DQB1* | 1 | 0.999999 |
|  | *HLA-DRB1* | 1 | 0.999982 |
|  | *ICAM1* | 1 | 0.999999 |
|  | *IFNG* | 1 | 0.999911 |
|  | *IFNGR1* | 1 | 0.999909 |
|  | *IGSF6* | 1 | 0.99976 |
|  | *IL10* | 3 | 0.999691 |
|  | *IL1B* | 1 | 0.998919 |
|  | *IL1R1* | 1 | 0.999993 |
|  | *IL1RN* | 4 | 0.998734 |
|  | *IL4R* | 1 | 0.999995 |
|  | *IL5* | 1 | 0.999897 |
|  | *ITGAL* | 4 | 0.998198 |
|  | *ITGB7* | 6 | 0.996454 |
|  | *ITPA* | 23 | 0.903318 |
|  | *LMAN1* | 9 | 0.978005 |
|  | *MLH1* | 38 | 0.911832 |
|  | *PLAT* | 36 | 0.913315 |
|  | *PTGS2* | 34 | 0.964567 |
|  | *SLC11A1* | 3 | 0.996377 |
|  | *TGFB1* | 1 | 0.999993 |
|  | *TLR4* | 1 | 0.999883 |
|  | *TNF* | 1 | 0.999993 |
|  | *TPMT* | 28 | 0.942422 |
| **Atherosclerosis** | *ACE* | 2 | 0.995143 |
|  | *AGTR1* | 7 | 0.971391 |
|  | *ALOX5* | 1 | 0.999706 |
|  | *APOA4* | 1 | 0.999982 |
|  | *APOB* | 10 | 0.989482 |
|  | *APOE* | 1 | 0.999909 |
|  | *CBS* | 1 | 0.997812 |
|  | *CD14* | 14 | 0.969588 |
|  | *CETP* | 26 | 0.902629 |
|  | *CMA1* | 37 | 0.848771 |
|  | *CX3CR1* | 27 | 0.956042 |
|  | *CXCL12* | 49 | 0.812775 |
|  | *CYBA* | 3 | 0.990872 |
|  | *CYP11B2* | 6 | 0.989482 |
|  | *CYP2E1* | 3 | 0.992903 |
|  | *FCGR2A* | 37 | 0.904041 |
|  | *GSTM1* | 2 | 0.989568 |
|  | *GSTT1* | 38 | 0.870395 |
|  | *HFE* | 9 | 0.977422 |
|  | *IL6* | 2 | 0.991156 |
|  | *INS* | 11 | 0.956868 |
|  | *LRP1* | 5 | 0.989643 |
|  | *MMP13* | 12 | 0.929654 |
|  | *MMP3* | 2 | 0.996813 |
|  | *MPO* | 40 | 0.722124 |
|  | *MTHFR* | 6 | 0.984079 |
|  | *NOS3* | 1 | 0.999582 |
|  | *PCK1* | 4 | 0.987769 |
|  | *PON1* | 2 | 0.997054 |
|  | *PON2* | 1 | 0.998664 |
|  | *SELE* | 20 | 0.945754 |
|  | *SERPINE1* | 3 | 0.990551 |
|  | *SREBF1* | 24 | 0.941442 |
|  | *TLR4* | 2 | 0.991703 |
|  | *TNF* | 2 | 0.997728 |
| **Ulcerative colitis** | *ABCB1* | 21 | 0.622875 |
|  | *BAT1* | 25 | 0.534636 |
|  | *BAT2* | 49 | 0.337805 |
|  | *NOD2* | 3 | 0.999274 |
|  | *CCL11* | 1 | 0.999989 |
|  | *CCL24* | 3 | 0.996734 |
|  | *CCL26* | 1 | 0.999095 |
|  | *CD14* | 1 | 0.998657 |
|  | *CTLA4* | 3 | 0.942175 |
|  | *HLA-DRA* | 5 | 0.994454 |
|  | *HLA-DRB1* | 1 | 0.999992 |
|  | *IL10* | 1 | 0.99999 |
|  | *IL11* | 4 | 0.979006 |
|  | *IL1B* | 1 | 0.999999 |
|  | *IL1R1* | 1 | 0.999796 |
|  | *IL1RN* | 2 | 0.999744 |
|  | *IL4* | 1 | 0.999998 |
|  | *KRAS* | 4 | 0.839219 |
|  | *LTA* | 1 | 0.999993 |
|  | *MICA* | 1 | 0.986043 |
|  | *MIF* | 5 | 0.898065 |
|  | *MLH1* | 32 | 0.431276 |
|  | *MMP1* | 56 | 0.305752 |
|  | *MMP3* | 3 | 0.94851 |
|  | *MUC3A* | 50 | 0.269077 |
|  | *NFKB1* | 1 | 0.999737 |
|  | *NFKBIL1* | 2 | 0.930738 |
|  | *NOS1* | 19 | 0.632512 |
|  | *PLA2G7* | 37 | 0.401783 |
|  | *TLR4* | 1 | 0.999928 |
|  | *TNF* | 1 | 1 |
|  | *TP53* | 8 | 0.676347 |
|  | *TPMT* | 27 | 0.560529 |
|  | *TRA@* | 2 | 0.999393 |
| **Hypercholesterolaemia** | *ABCA1* | 1 | 1 |
|  | *ABCB1* | 1 | 0.99992 |
|  | *ABCG5* | 1 | 0.999166 |
|  | *ABCG8* | 7 | 0.816268 |
|  | *APOA1* | 1 | 1 |
|  | *APOA4* | 1 | 0.999945 |
|  | *APOA5* | 2 | 0.999998 |
|  | *APOB* | 1 | 0.99999 |
|  | *APOC3* | 1 | 0.999992 |
|  | *APOE* | 1 | 1 |
|  | *CETP* | 1 | 0.999979 |
|  | *CYBA* | 3 | 0.889739 |
|  | *CYP1A1* | 1 | 0.999974 |
|  | *CYP2C19* | 1 | 1 |
|  | *CYP2C9* | 1 | 1 |
|  | *CYP2D6* | 2 | 1 |
|  | *CYP3A4* | 1 | 1 |
|  | *CYP3A5* | 1 | 1 |
|  | *CYP7A1* | 1 | 0.999817 |
|  | *FABP2* | 7 | 0.91853 |
|  | *ITIH4* | 2 | 0.996293 |
|  | *LDLR* | 1 | 0.999993 |
|  | *LIPC* | 2 | 0.99999 |
|  | *LPL* | 1 | 1 |
|  | *NAT2* | 31 | 0.46093 |
|  | *PCSK9* | 1 | 0.994348 |
|  | *PON1* | 2 | 0.999975 |
|  | *PON2* | 1 | 0.999489 |
|  | *SCARB1* | 1 | 0.999787 |
|  | *SREBF1* | 2 | 0.992694 |
|  | *SREBF2* | 4 | 0.928397 |
|  | *TLR4* | 11 | 0.727075 |
| **Endometrial carcinoma** | *ADRB3* | 22 | 0.654006 |
|  | *AR* | 1 | 0.999996 |
|  | *BRAF* | 2 | 0.971971 |
|  | *BRCA1* | 1 | 0.999999 |
|  | *CCND1* | 4 | 0.999706 |
|  | *COMT* | 1 | 0.984199 |
|  | *CYP17A1* | 2 | 0.999871 |
|  | *CYP19A1* | 1 | 0.999998 |
|  | *CYP1A1* | 1 | 0.999892 |
|  | *CYP1A2* | 2 | 0.999906 |
|  | *CYP1B1* | 1 | 0.999989 |
|  | *ERCC1* | 1 | 0.999556 |
|  | *ERCC2* | 1 | 1 |
|  | *ERCC4* | 1 | 0.996852 |
|  | *ERCC5* | 1 | 0.998178 |
|  | *ESR1* | 1 | 0.999989 |
|  | *ESR2* | 1 | 0.999243 |
|  | *KRAS* | 6 | 0.92993 |
|  | *MLH1* | 1 | 1 |
|  | *MSH2* | 1 | 0.999995 |
|  | *MSH6* | 1 | 0.999978 |
|  | *MUC1* | 41 | 0.351027 |
|  | *PAK3* | 28 | 0.482752 |
|  | *PGR* | 1 | 0.999933 |
|  | *PTEN* | 9 | 0.896788 |
|  | *TP53* | 1 | 0.999982 |
|  | *TP73* | 7 | 0.786815 |
|  | *UGT1A1* | 57 | 0.277167 |
|  | *XPA* | 1 | 0.999798 |
|  | *XPC* | 1 | 0.999831 |
|  | *XRCC1* | 1 | 0.999991 |
|  | *XRCC3* | 1 | 0.999027 |
|  | *XRCC5* | 1 | 0.999974 |
| **Migraine** | *AR* | 1 | 0.999604 |
|  | *CACNA1A* | 7 | 0.857373 |
|  | *CTLA4* | 59 | 0.252297 |
|  | *DBH* | 1 | 0.978144 |
|  | *DIDO1* | 43 | 0.268983 |
|  | *DRD1* | 1 | 0.999998 |
|  | *DRD3* | 1 | 0.999725 |
|  | *DRD4* | 1 | 0.999999 |
|  | *DRD5* | 1 | 0.999978 |
|  | *EDNRA* | 2 | 0.970762 |
|  | *EDNRB* | 1 | 0.999353 |
|  | *EFHC1* | 2 | 0.966193 |
|  | *ESR1* | 1 | 0.999973 |
|  | *F8* | 14 | 0.578104 |
|  | *HTR2A* | 1 | 0.999828 |
|  | *IL1A* | 5 | 0.897598 |
|  | *INSR* | 1 | 0.995402 |
|  | *KCNN3* | 33 | 0.396728 |
|  | *MA* | 86 | 0 |
|  | *MAOA* | 1 | 0.999661 |
|  | *MAOB* | 2 | 0.974115 |
|  | *MEP1A* | 3 | 0.952115 |
|  | *MTHFD1* | 10 | 0.779662 |
|  | *MTHFR* | 16 | 0.659532 |
|  | *NOS1* | 3 | 0.964018 |
|  | *NOS2A* | 1 | 0.999978 |
|  | *PGR* | 2 | 0.980038 |
|  | *RHAG* | 1 | 0.981596 |
|  | *SLC25A27* | 26 | 0.524813 |
|  | *SLC6A4* | 1 | 0.989018 |
|  | *TNF* | 1 | 0.986213 |
|  | *TNFRSF21* | 25 | 0.593299 |
|  | *TYMS* | 45 | 0.284864 |
| **Pancreatitis** | *ADH1B* | 2 | 0.996071 |
|  | *ALDH2* | 1 | 0.993834 |
|  | *CAT* | 70 | 0.252752 |
|  | *CD14* | 1 | 0.977734 |
|  | *CFTR* | 1 | 0.999914 |
|  | *GSTM1* | 1 | 0.999997 |
|  | *GSTM3* | 1 | 0.999785 |
|  | *GSTP1* | 2 | 0.999343 |
|  | *GSTT1* | 1 | 0.999997 |
|  | *HLA-A* | 1 | 0.999992 |
|  | *HLA-B* | 1 | 0.999999 |
|  | *HLA-C* | 1 | 1 |
|  | *HLA-DPB1* | 1 | 1 |
|  | *HLA-DQB1* | 1 | 0.999998 |
|  | *HLA-DRB1* | 1 | 0.999997 |
|  | *HRAS* | 38 | 0.363793 |
|  | *HSPA1B* | 4 | 0.940119 |
|  | *IFNG* | 1 | 0.999096 |
|  | *IL10* | 1 | 0.999773 |
|  | *KRT8* | 18 | 0.674613 |
|  | *MGST1* | 2 | 0.999351 |
|  | *PON1* | 51 | 0.435736 |
|  | *PRSS1* | 2 | 0.999844 |
|  | *SOD2* | 10 | 0.756892 |
|  | *SPINK1* | 2 | 0.999628 |
|  | *TGFB1* | 1 | 0.99968 |
|  | *TNF* | 1 | 0.999512 |
|  | *UGT1A1* | 1 | 0.999966 |
|  | *UGT1A6* | 1 | 0.999977 |
|  | *UGT1A7* | 1 | 0.999875 |
|  | *UGT1A8* | 2 | 0.999939 |
| **Systemic scleroderma** | *ACE* | 2 | 0.992313 |
|  | *CCL2* | 1 | 0.999154 |
|  | *CD19* | 5 | 0.996977 |
|  | *COL1A2* | 2 | 0.999957 |
|  | *COL3A1* | 1 | 0.999939 |
|  | *CTLA4* | 1 | 0.999543 |
|  | *CYBA* | 11 | 0.915665 |
|  | *CYP2C19* | 40 | 0.688968 |
|  | *CYP2E1* | 62 | 0.578951 |
|  | *FBN1* | 1 | 0.999992 |
|  | *FN1* | 2 | 0.999572 |
|  | *HLA-A* | 1 | 0.999999 |
|  | *HLA-DPB1* | 1 | 1 |
|  | *HLA-DQA1* | 1 | 1 |
|  | *HLA-DQB1* | 1 | 1 |
|  | *HLA-DRB1* | 1 | 1 |
|  | *HLA-DRB3* | 1 | 1 |
|  | *HLA-DRB4* | 1 | 0.999999 |
|  | *HLA-DRB5* | 1 | 0.999999 |
|  | *IL1A* | 2 | 0.993534 |
|  | *MMP1* | 3 | 0.994669 |
|  | *NOS3* | 36 | 0.690622 |
|  | *PDGFB* | 1 | 0.995155 |
|  | *PTPRC* | 1 | 0.995126 |
|  | *SPARC* | 1 | 0.99922 |
|  | *TAP1* | 1 | 0.999213 |
|  | *TAP2* | 2 | 0.995239 |
|  | *TERC* | 61 | 0.212679 |
|  | *TGFB1* | 1 | 0.999998 |
|  | *TNF* | 1 | 0.999999 |
|  | *TNFRSF1B* | 1 | 0.9992 |
| **Cirrhosis** | *ADH1B* | 1 | 0.999572 |
|  | *ADH1C* | 1 | 0.999995 |
|  | *ALDH2* | 1 | 0.999554 |
|  | *C3* | 2 | 0.997017 |
|  | *COMT* | 2 | 0.982333 |
|  | *CYP17A1* | 36 | 0.463252 |
|  | *CYP2E1* | 1 | 0.999894 |
|  | *EPHX1* | 12 | 0.962977 |
|  | *GSTP1* | 13 | 0.729424 |
|  | *HFE* | 37 | 0.396952 |
|  | *HLA-DQB1* | 1 | 0.999188 |
|  | *HLA-DRB1* | 2 | 0.994799 |
|  | *HMOX1* | 1 | 0.991707 |
|  | *IL10RA* | 20 | 0.807454 |
|  | *IL1A* | 3 | 0.999628 |
|  | *IL1B* | 1 | 0.999903 |
|  | *IL6* | 2 | 0.999998 |
|  | *IL8* | 1 | 0.999968 |
|  | *KIF21A* | 82 | 0 |
|  | *LTA* | 1 | 0.999989 |
|  | *MPO* | 32 | 0.661053 |
|  | *NOS2A* | 3 | 0.977127 |
|  | *PHOX2A* | 51 | 0.259637 |
|  | *SERPINA1* | 11 | 0.763458 |
|  | *SOD2* | 2 | 0.998112 |
|  | *SRD5A2* | 16 | 0.884359 |
|  | *TFRC* | 27 | 0.62471 |
|  | *TGFB1* | 1 | 0.999871 |
|  | *TNF* | 1 | 0.999975 |
|  | *TNFRSF1A* | 1 | 0.999744 |
