# Supplementary material for: Improved human disease candidate gene prioritization using mouse phenotype
Source: BMC Bioinformatics. 2007 Oct 16;8:392. doi: 10.1186/1471-2105-8-392 (PMC2194797; doi:10.1186/1471-2105-8-392)
Supplement: Additional file 3 — List and ranking of "target" genes in locus-region cross-validation using different feature sets. This file has the details of the ranking of the "target" genes in locus-region cross-validation using different gene feature sets. When MP or PubMed annotations were excluded in the prioritization, the prioritization performance dropped significantly. [file 1471-2105-8-392-S3.doc]

**Additional file 3:** Ranking of the “target” genes in locus-region cross-validation using different gene feature sets. When MP (mammalian phenotype) or PubMed annotations were excluded in the prioritization, the prioritization performance dropped significantly.

| **Disease name** | **Gene symbol** | **Rank using all features** | **Without MP** | **Without PubMed** | **Number of test genes** |
| --- | --- | --- | --- | --- | --- |
| **Glaucoma, primary open angle, juvenile-onset** | *CYP1B1* | 2 | 3 | 10 | 60 |
|  | *LMX1B* | 2 | 140 | 2 | 175 |
|  | *MYOC* | 1 | 1 | 23 | 89 |
| **Adrenoleukodystrophy, autosomal neonatal form** | *PEX1* | 2 | 2 | 37 | 88 |
|  | *PEX10* | 1 | 1 | 1 | 96 |
|  | *PEX13* | 1 | 1 | 1 | 55 |
|  | *PEX5* | 1 | 1 | 1 | 171 |
| **Lupus erythematosus, systemic** | *C1QA* | 1 | 10 | 16 | 190 |
|  | *DNASE1* | 2 | 36 | 2 | 175 |
|  | *FCGR2A* | 4 | 3 | 70 | 223 |
| **Meningioma, familial** | *ALPL* | 10 | 14 | 9 | 187 |
|  | *AP1B1* | 39 | 34 | 35 | 140 |
|  | *PTEN* | 1 | 1 | 1 | 69 |
| **Parkinson’s disease** | *PARK7* | 1 | 1 | 5 | 145 |
|  | *SNCA* | 1 | 1 | 1 | 50 |
|  | *UCHL1* | 1 | 1 | 1 | 53 |
| **Retinitis pigmentosa** | *CRB1* | 2 | 9 | 2 | 84 |
|  | *IMPDH1* | 15 | 42 | 14 | 79 |
|  | *MERTK* | 3 | 14 | 3 | 67 |
|  | *PRPF3* | 1 | 1 | 40 | 250 |
|  | *PRPF31* | 3 | 3 | 50 | 341 |
|  | *PRPF8* | 5 | 3 | 85 | 183 |
|  | *RHO* | 1 | 1 | 1 | 106 |
|  | *RP1* | 1 | 1 | 1 | 41 |
|  | *TULP1* | 1 | 2 | 2 | 275 |
| **Bardet Biedl** | *BBS1* | 2 | 2 | 3 | 309 |
|  | *BBS2* | 1 | 1 | 1 | 77 |
|  | *BBS4* | 1 | 1 | 2 | 126 |
|  | *DPP3* | 4 | 4 | 4 | 309 |
|  | *MKKS* | 1 | 1 | 1 | 66 |
| **Epidermolysis bullosa letalis** | *ITGB4* | 1 | 1 | 1 | 184 |
|  | *LAMA3* | 1 | 1 | 1 | 47 |
|  | *LAMC2* | 1 | 1 | 1 | 67 |
| **Inflammatory bowel disease** | *IL18* | 1 | 1 | 1 | 116 |
|  | *IL18BP* | 1 | 1 | 1 | 219 |
|  | *IL1B* | 3 | 2 | 2 | 65 |
|  | *IL1R1* | 1 | 2 | 1 | 89 |
|  | *NOD2* | 1 | 2 | 2 | 84 |
| **Charcot-marie-tooth disease, demyelinating types 1A-1F** | *EGR2* | 1 | 6 | 1 | 50 |
|  | *MPZ* | 1 | 1 | 1 | 231 |
|  | *PMP22* | 1 | 1 | 1 | 115 |
| **Prostate cancer** | *CD82* | 55 | 55 | 39 | 63 |
|  | *ELAC2* | 12 | 11 | 9 | 167 |
|  | *KLF6* | 13 | 11 | 10 | 41 |
|  | *MAD1L1* | 16 | 15 | 17 | 55 |
|  | *MXI1* | 14 | 43 | 14 | 63 |
|  | *PTEN* | 1 | 1 | 2 | 69 |
| **Thyroid carcinoma, papillary** | *GOLGA5* | 2 | 2 | 4 | 86 |
|  | *NCOA4* | 3 | 3 | 5 | 48 |
|  | *PCM1* | 17 | 16 | 44 | 84 |
|  | *PRKAR1A* | 3 | 3 | 13 | 131 |
|  | *RET* | 1 | 1 | 1 | 51 |
|  | *TFG* | 11 | 10 | 13 | 41 |
|  | *TPM3* | 65 | 69 | 135 | 322 |
|  | *TPR* | 15 | 14 | 28 | 66 |
|  | *TRIM24* | 32 | 30 | 23 | 91 |
| **Diabetes mellitus, noninsulin-dependent** | *GPD2* | 19 | 21 | 16 | 46 |
|  | *HNF4A* | 1 | 1 | 1 | 124 |
|  | *MAPK8IP1* | 31 | 24 | 30 | 63 |
|  | *NEUROD1* | 2 | 4 | 8 | 52 |
|  | *SLC2A2* | 1 | 1 | 1 | 34 |
|  | *SLC2A4* | 2 | 3 | 2 | 209 |
|  | *TCF1* | 1 | 1 | 1 | 101 |
|  | *TCF2* | 1 | 1 | 1 | 269 |
| **Maturity-onset diabetes of the young** | *GCK* | 1 | 1 | 1 | 68 |
|  | *HNF4A* | 1 | 1 | 1 | 124 |
|  | *NEUROD1* | 1 | 1 | 1 | 52 |
|  | *PDX1* | 1 | 1 | 1 | 62 |
|  | *TCF1* | 1 | 1 | 1 | 101 |
|  | *TCF2* | 1 | 1 | 1 | 269 |
| **Muscular dystrophy, limb-girdle, autosomal recessive** | *CAPN3* | 2 | 2 | 2 | 117 |
|  | *DYSF* | 1 | 2 | 2 | 103 |
|  | *FKRP* | 134 | 111 | 121 | 398 |
|  | *GANC* | 2 | 2 | 29 | 118 |
|  | *SGCA* | 1 | 1 | 1 | 177 |
|  | *SGCB* | 1 | 1 | 1 | 49 |
|  | *SGCG* | 1 | 1 | 1 | 59 |
|  | *TRIM32* | 58 | 48 | 33 | 94 |
| **Acute myelogenous leukemia, familial** | *EGFR* | 2 | 17 | 3 | 31 |
|  | *GMPS* | 2 | 2 | 31 | 63 |
|  | *MLL* | 3 | 3 | 20 | 148 |
|  | *MLLT1* | 16 | 16 | 199 | 343 |
| **Long-segment Hirschsprung disease** | *EDN3* | 1 | 1 | 1 | 104 |
|  | *EDNRB* | 1 | 1 | 1 | 21 |
|  | *GDNF* | 1 | 1 | 1 | 69 |
|  | *RET* | 1 | 1 | 1 | 51 |
|  | *SOX10* | 2 | 5 | 14 | 186 |
| **Familial adenomatous polyposis** | *APC* | 1 | 1 | 2 | 37 |
|  | *PLA2G2D* | 3 | 3 | 2 | 179 |
|  | *PLA2G4A* | 2 | 2 | 2 | 61 |
|  | *PTGS2* | 2 | 2 | 2 | 62 |
| **Alzheimer’s disease** | *APOE* | 1 | 1 | 1 | 393 |
|  | *APP* | 1 | 1 | 1 | 49 |
|  | *PSEN1* | 1 | 1 | 1 | 105 |
|  | *PSEN2* | 1 | 1 | 1 | 96 |
| **Basal cell carcinoma** | *PTCH2* | 1 | 1 | 1 | 156 |
|  | *RASA1* | 8 | 12 | 9 | 35 |
|  | *SMO* | 1 | 1 | 1 | 76 |
| **Breast cancer** | *BRCA1* | 1 | 1 | 1 | 305 |
|  | *BRCA2* | 1 | 1 | 1 | 62 |
|  | *BRIP1* | 9 | 8 | 9 | 117 |
|  | *ESR1* | 1 | 1 | 1 | 62 |
|  | *PPM1D* | 24 | 39 | 23 | 108 |
|  | *RB1CC1* | 8 | 10 | 8 | 36 |
|  | *SLC22A18* | 167 | 163 | 97 | 206 |
|  | *TP53* | 1 | 1 | 1 | 209 |
|  | *TSG101* | 1 | 2 | 2 | 61 |
| **Cardiomyopathy, familial hypertrophic** | *ACTC1* | 1 | 1 | 1 | 87 |
|  | *MYH7* | 1 | 1 | 81 | 134 |
|  | *MYL2* | 2 | 2 | 2 | 108 |
|  | *MYLK2* | 1 | 1 | 1 | 139 |
|  | *PRKAG2* | 23 | 23 | 20 | 78 |
|  | *TNNI3* | 1 | 1 | 1 | 324 |
|  | *TNNT2* | 1 | 1 | 1 | 116 |
|  | *TPM1* | 1 | 1 | 1 | 91 |
|  | *TTN* | 1 | 1 | 1 | 60 |
| **Colorectal cancer, hereditary nonpolyposis** | *MLH1* | 1 | 1 | 1 | 79 |
|  | *MSH2* | 2 | 2 | 2 | 53 |
|  | *MSH6* | 2 | 2 | 2 | 56 |
|  | *PMS1* | 1 | 1 | 1 | 40 |
|  | *PMS2* | 1 | 1 | 1 | 60 |
|  | *TGFBR2* | 2 | 4 | 4 | 46 |
| **Epiphyseal dysplasia, multiple types 1-5** | *COL2A1* | 1 | 1 | 1 | 181 |
|  | *COL9A1* | 1 | 1 | 1 | 30 |
|  | *COL9A2* | 1 | 1 | 1 | 182 |
|  | *COL9A3* | 1 | 1 | 1 | 96 |
|  | *COMP* | 1 | 1 | 1 | 229 |
|  | *MATN3* | 1 | 1 | 1 | 74 |
| **Gastric cancer** | *APC* | 1 | 1 | 1 | 37 |
|  | *CASP10* | 26 | 25 | 23 | 75 |
|  | *CDH1* | 2 | 2 | 4 | 135 |
|  | *FGFR2* | 6 | 11 | 4 | 69 |
| **Hypertension** | *AGTR1* | 1 | 1 | 1 | 61 |
|  | *HSD11B2* | 1 | 1 | 1 | 123 |
|  | *NR3C2* | 1 | 1 | 2 | 55 |
|  | *PNMT* | 212 | 212 | 165 | 267 |
|  | *PTGIS* | 17 | 83 | 16 | 115 |
| **Leber congenital amaurosis** | *AIPL1* | 2 | 2 | 3 | 205 |
|  | *CRB1* | 1 | 1 | 1 | 84 |
|  | *GUCY2D* | 2 | 2 | 9 | 208 |
|  | *RPE65* | 1 | 1 | 1 | 61 |
|  | *RPGRIP1* | 1 | 1 | 3 | 130 |
| **Ovarian carcinoma** | *AKT2* | 42 | 48 | 37 | 328 |
|  | *CDH1* | 3 | 3 | 4 | 135 |
|  | *CTNNB1* | 1 | 1 | 1 | 129 |
|  | *RRAS2* | 4 | 4 | 3 | 104 |
| **Cystic fibrosis** | *CFTR* | 1 | 1 | 1 | 42 |
|  | *MBL2* | 10 | 9 | 10 | 44 |
|  | *NOS1* | 9 | 39 | 8 | 141 |
|  | *TGFB1* | 3 | 9 | 30 | 349 |
