# Supplementary material for: Improved human disease candidate gene prioritization using mouse phenotype
Source: BMC Bioinformatics. 2007 Oct 16;8:392. doi: 10.1186/1471-2105-8-392 (PMC2194797; doi:10.1186/1471-2105-8-392)
Supplement: Additional file 4 — Comparison of relative rankings of "target" genes of congenital heart disease using SUSPECTS, ENDEAVOUR and ToppGene. The data provided represent the ranking results of "target" genes of congenital heart disease using SUSPECTS, ENDEAVOUR and ToppGene applications. [file 1471-2105-8-392-S4.doc]

**Additional file 4:** The ranking results of “target” genes of congenital heart disease (CHD) using SUSPECTS, ENDEAVOUR and ToppGene applications. The number before slash is the rank of the “target” gene, and the number after is the number of random genes in the corresponding test set. The sizes of the test sets may be slightly different for the three applications because some of these genes are not found in a particular application.

|  | **Gene symbol** | **SUSPECTS** | **ENDEAVOUR** | **ToppGene** |
| --- | --- | --- | --- | --- |
| 1 | *ADD1* | 3/24 | 4/24 | 23/24 |
| 2 | *CITED2* | 9/19 | 1/19 | 1/19 |
| 3 | *DTNA* | 7/18 | 5/18 | 5/18 |
| 4 | *CKM* | 9/26 | 6/26 | 5/26 |
| 5 | *GATA4* | 1/19 | 1/19 | 1/19 |
| 6 | *GJA1* | 1/15 | 2/15 | 1/15 |
| 7 | *HAND1* | 1/22 | 1/22 | 1/22 |
| 8 | *HAND2* | 4/29 | 1/28 | 1/29 |
| 9 | *HEY2* | 10/23 | 6/22 | 1/23 |
| 10 | *HOXC4* | 11/15 | 2/15 | 2/15 |
| 11 | *HOXC5* | 10/22 | 2/22 | 4/22 |
| 12 | *ITGB3* | 1/26 | 16/26 | 14/26 |
| 13 | *JARID2* | 10/25 | 6/25 | 1/25 |
| 14 | *MTHFD1* | 1/17 | 6/17 | 14/17 |
| 15 | *MTHFR* | 2/20 | 4/19 | 3/20 |
| 16 | *MTRR* | 1/20 | 1/20 | 7/20 |
| 17 | *NKX2-5* | 1/17 | 1/15 | 1/17 |
| 18 | *NOS3* | 4/17 | 3/16 | 1/17 |
| 19 | *NPPA* | 2/19 | 3/18 | 1/19 |
| 20 | *NPPB* | 1/23 | 2/21 | 1/23 |
| 21 | *RFC1* | 13/20 | 14/19 | 3/20 |
| 22 | *SALL4* | 4/19 | 1/17 | 1/19 |
| 23 | *TBX1* | 1/20 | 3/20 | 2/20 |
| 24 | *TBX5* | 2/20 | 1/16 | 1/20 |
| 25 | *TBX20* | 7/19 | 1/18 | 1/19 |
| 26 | *TGFB1* | 4/15 | 1/15 | 1/15 |
| 27 | *ZFPM2* | 13/17 | 1/16 | 1/17 |
| 28 | *ZIC3* | 6/24 | 1/21 | 1/24 |
