# Supplementary material for: Improved human disease candidate gene prioritization using mouse phenotype
Source: BMC Bioinformatics. 2007 Oct 16;8:392. doi: 10.1186/1471-2105-8-392 (PMC2194797; doi:10.1186/1471-2105-8-392)
Supplement: Additional file 5 — Comparison of relative rankings of "target" genes of diabetic retinopathy using SUSPECTS, ENDEAVOUR and ToppGene. The data provided represent the ranking results of "target" genes of diabetic retinopathy using SUSPECTS, ENDEAVOUR and ToppGene applications. [file 1471-2105-8-392-S5.doc]

**Additional file 5:** The ranking results of “target” genes of diabetic retinopathy (DR) using SUSPECTS, ENDEAVOUR and ToppGene applications. The number before slash is the rank of the “target” gene, and the number after is the number of neighboring genes in the corresponding locus region. The sizes of the test sets may be slightly different for the three applications because some of the genes are not found in a particular application.

|  | **Gene symbol** | **SUSPECTS** | **ENDEAVOUR** | **ToppGene** |
| --- | --- | --- | --- | --- |
| 1 | *ACE* | 1/51 | 15/56 | 1/58 |
| 2 | *ADRB3* | 34/39 | 6/43 | 8/43 |
| 3 | *AGT* | 1/54 | 1/64 | 1/65 |
| 4 | *AGTR2* | 2/36 | 4/39 | 1/40 |
| 5 | *AKR1B1* | 7/29 | 7/35 | 11/35 |
| 6 | *APOE* | 1/204 | 40/227 | 4/236 |
| 7 | *AR* | 27/40 | 10/43 | 6/43 |
| 8 | *CMA1* | 20/104 | 75/110 | 5/116 |
| 9 | *EDN1* | 1/34 | 3/28 | 1/36 |
| 10 | *GNB3* | 3/100 | 39/107 | 5/115 |
| 11 | *HFE* | 1/150 | 5/120 | 45/150 |
| 12 | *HLA-DPB1* | 1/185 | 6/200 | 1/205 |
| 13 | *HLA-DRB1* | 12/192 | 4/210 | 2/222 |
| 14 | *ICAM1* | 7/178 | 6/197 | 1/204 |
| 15 | *ITGA2B* | 10/148 | 47/165 | 47/171 |
| 16 | *ITGB2* | 1/75 | 1/77 | 2/90 |
| 17 | *LTA* | 27/180 | 34/202 | 11/220 |
| 18 | *NOS2A* | 4/65 | 1/78 | 14/80 |
| 19 | *NOS3* | 5/38 | 1/43 | 1/47 |
| 20 | *NPY* | 1/37 | 2/41 | 7/41 |
| 21 | *PECAM1* | 35/48 | 3/54 | 1/56 |
| 22 | *PON1* | 13/56 | 1/68 | 7/70 |
| 23 | *RAGE* | 33/49 | 19/55 | 11/56 |
| 24 | *SELE* | 5/47 | 2/52 | 2/53 |
| 25 | *SERPINE1* | 2/75 | 1/94 | 2/97 |
| 26 | *TIMP3* | 3/85 | 7/93 | 5/95 |
| 27 | *TNF* | 17/180 | 19/202 | 8/203 |
