# Supplementary material for: Improved human disease candidate gene prioritization using mouse phenotype
Source: BMC Bioinformatics. 2007 Oct 16;8:392. doi: 10.1186/1471-2105-8-392 (PMC2194797; doi:10.1186/1471-2105-8-392)
Supplement: Additional file 6 — Pairwise Pearson correlation test result of the p-values of all the 7 features used for candidate gene prioritization. This figure shows the pairwise Pearson correlation test result of the p-values of all the features used for candidate gene prioritization. [file 1471-2105-8-392-S6.doc]

**Additional file 6:** Pair wise Pearson correlation test result of the *p-values* of all the features. The *p-value* of GO was created by taking the p-value of the average of GO:MF and GO:BP scores from the random sample. The result showed that the correlations of *p-values* from different features were low and so the independent requirement of Fisher’s meta-analysis was satisfied.
